# The significance of NAD + metabolites and nicotinamide N-methyltransferase in chronic kidney disease

**DOI:** 10.1038/s41598-022-10476-6

**Published:** 2022-04-16

**Authors:** Rina Takahashi, Takeshi Kanda, Motoaki Komatsu, Tomoaki Itoh, Hitoshi Minakuchi, Hidenori Urai, Tomohiro Kuroita, Shuhei Shigaki, Tasuku Tsukamoto, Naoko Higuchi, Minoru Ikeda, Risa Yamanaka, Norito Yoshimura, Takashi Ono, Hideo Yukioka, Kazuhiro Hasegawa, Hirobumi Tokuyama, Shu Wakino, Hiroshi Itoh

**Affiliations:** 1 Department of Internal Medicine, School of Medicine, Keio University, 35, Shinanomachi, Shinjuku-ku, Tokyo, 160-8582 Japan; 2 Biomarker Research and Development Department, Shionogi & Co., Ltd., 3-1-1, Futaba-cho, Toyonaka-shi, Osaka, 561-0825 Japan; 3 Drug Discovery and Disease Research Laboratory, Shionogi & Co., Ltd., 3-1-1, Futaba-cho, Toyonaka-shi, Osaka, 561-0825 Japan

**Keywords:** Medical research, Nephrology

## Abstract

Dysregulation of nicotinamide adenine dinucleotide (NAD +) metabolism contributes to the initiation and progression of age-associated diseases, including chronic kidney disease (CKD). Nicotinamide N-methyltransferase (NNMT), a nicotinamide (NAM) metabolizing enzyme, regulates both NAD + and methionine metabolism. Although NNMT is expressed abundantly in the kidney, its role in CKD and renal fibrosis remains unclear. We generated NNMT-deficient mice and a unilateral ureter obstruction (UUO) model and conducted two clinical studies on human CKD to investigate the role of NNMT in CKD and fibrosis. In UUO, renal NNMT expression and the degraded metabolites of NAM increased, while NAD + and NAD + precursors decreased. NNMT deficiency ameliorated renal fibrosis; mechanistically, it (1) increased the DNA methylation of connective tissue growth factor (CTGF), and (2) improved renal inflammation by increasing renal NAD + and Sirt1 and decreasing NF-κB acetylation. In humans, along with CKD progression, a trend toward a decrease in serum NAD + precursors was observed, while the final NAD + metabolites were accumulated, and the level of eGFR was an independent variable for serum NAM. In addition, NNMT was highly expressed in fibrotic areas of human kidney tissues. In conclusion, increased renal NNMT expression induces NAD + and methionine metabolism perturbation and contributes to renal fibrosis.

## Introduction

Chronic kidney disease (CKD) patients are at high risk for end-stage renal failure and cardiovascular diseases. Further, this non-communicable disease is a significant global public health burden due to its increasing prevalence and mortality^1,2^. Multiple approaches, such as renin-angiotensin system blockades, statins, and glucose-lowering medications, are used to treat CKD. These approaches reduce CKD progression in diabetic patients with albuminuria. However, they also increase the prevalence of reduced estimated glomerular filtration rate (eGFR), especially in elderly patients^3^. Therefore, there is an urgent need to develop new strategies to prevent CKD.

**Figure 1:**
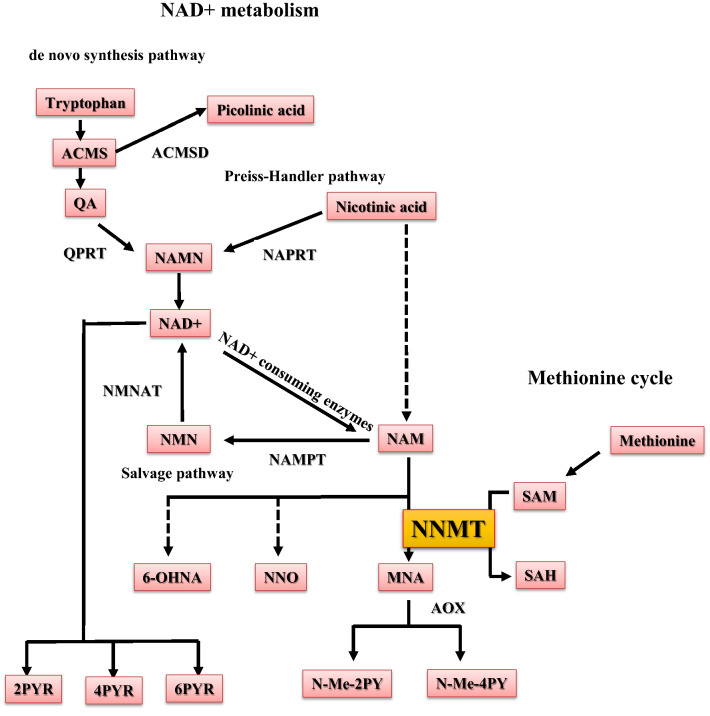
NNMT regulates NAD + and methionine metabolism. NAD + -consuming enzymes (Sirtuins, PARPs, cADPR synthases) degrade NAD + to NAM. NAM is converted to NMN by NAMPT for reuse in NAD + biosynthesis (salvage pathway). Otherwise, NAM is metabolized to MNA by NNMT. NNMT uses SAM as a methyl donor and yields MNA and SAH from NAM. N-Me-2PY and N-Me-4PY are the major final NAM metabolites. In addition to the salvage pathway, there are two pathways of NAD + synthesis; the de novo pathway, in which NAD + is synthesized from tryptophan via alpha-amino-beta-carboxy-muconate-semialdehyde (ACMS), quinolinic acid (QA), and nicotinic acid mononucleotide (NAMN) by quinolinate phosphoribosyltransferase (QPRT), and the Preiss–Handler pathway, in which NAD + is synthesized from nicotinic acid (NA) via NAMN by nicotinate phosphoribosyltransferase (NAPRT). ACMS is degraded to picolinic acid by alpha-amino-beta-carboxy-muconate-semialdehyde decarboxylase (ACMSD). NAM, nicotinamide; NAD + , nicotinamide adenine dinucleotide; NMN, nicotinamide mononucleotide; PARP, poly ADP ribose polymerase; cADPR, cyclic ADP ribose; NAMPT, nicotinamide phosphoribosyltransferase; NNMT, nicotinamide N-methyltransferase; NMNAT, nicotinamide mononucleotide adenylyltransferase; MNA, 1-methylnicotinamide; NNO, nicotinamide-N-oxide; 6-OHNA, 6-hydroxynicotinic acid; N-Me-2PY, N-methyl-2-pyridone-5-carboxamide; N-Me-4PY, N-methyl-4-pyridone-3-carboxamide; SAM, S-adenosylmethionine; SAH, S-adenosylhomocysteine; ACMS, alpha-amino-beta-carboxy-muconate-semialdehyde; ACMSD, alpha-amino-beta-carboxy-muconate-semialdehyde decarboxylase; QA, quinolinic acid; QPRT, quinolinate phosphoribosyltransferase; NAMN, nicotinic acid mononucleotide; NA, nicotinic acid; NAPRT, nicotinate phosphoribosyltransferase.

Nicotinamide adenine dinucleotide (NAD +) is an essential cofactor for numerous regulatory proteins, including NAD + -dependent sirtuin, improving metabolic dysregulation and extending life span^4^. NAD + levels in multiple tissues are reduced along with aging and are correlated with reduced sirtuin activity^5^. Altered NAD + metabolism is related to aging and age-associated physiological dysfunction, such as diabetes and obesity^6^. Enhancing NAD + levels with NAD + precursors, such as nicotinamide (NAM) and nicotinamide riboside (NR), extends life span^5^ and ameliorates various metabolic abnormalities^7^. The kidney is a metabolically active organ dependent on tissue NAD + levels^8,9^. Therefore, NAD + metabolites are likely involved in the pathogenesis of CKD^9^. Previously, we reported that renal tubular sirtuin 1 (Sirt1) attenuates diabetic albuminuria through increased levels of NMN (nicotinamide mononucleotide)^10^. Additionally, N-methyl-2-pyridone-5-carboxamide (N-Me-2PY) and N-methyl-4-pyridone-3-carboxamide (N-Me-4PY), the final metabolites of NAD + metabolism (Fig. 1), are present at extremely high serum concentrations in CKD patients compared to healthy subjects; these are reported to be uremic toxins that can lead to renal cell damages^11,12^. These data indicate that NAD + metabolism plays a vital role in CKD progression. However, how NAD + metabolites influence CKD remains unclear.

Nicotinamide N-methyltransferase (NNMT) catalyzes the transfer of a methyl group from S-adenosylmethionine (SAM) to NAM, producing S-adenosylhomocysteine (SAH) and methyl nicotinamide (MNA). Subsequently, MNA is metabolized to N-Me-2PY and N-Me-4PY (Fig. 1). NNMT is involved in the development of age-associated non-communicable diseases, such as cancer and obesity, through the consumption of methyl donors, decreased NAD + content, and increased active NAD + metabolites^13,14^. This involvement of NNMT in age-associated diseases implies that the enzyme might also play an essential role in the initiation and progression of CKD. We previously reported that hepatic NNMT promotes liver inflammation and fibrosis by regulating NAD + and the methionine pathway through epigenetic changes^15^. NNMT is abundantly expressed in the kidney^8,16^ and regulates N-Me-2PY and N-Me-4PY levels^15^. N-Me-2PY, one of the end-products of NNMT, accumulates in CKD, and is a putative uremic toxin with an inhibitory potency toward poly (ADP ribose) polymerase (PARP). This nuclear enzyme is highly involved in various physiologic events, including the regulation of DNA repair^17^. Through these molecules, NNMT can be associated with the progression of CKD. However, the role of NNMT in chronic renal injury and fibrosis has not been elucidated.

Regardless of the cause of CKD, renal fibrosis is its typical final stage. Therefore, it is of great importance to understand the mechanism underlying the development of renal fibrosis. This study investigated the pathological significance of NAD + metabolites and NNMT in renal fibrosis using NNMT knockout mice. We also conducted two clinical studies to investigate the alteration of NAD + metabolites in human CKD and the role of NNMT in human renal fibrosis.

## Results

### Increased NNMT expression and NNMT-mediated NAD + metabolite fluctuations after UUO induction

**Figure 2:**
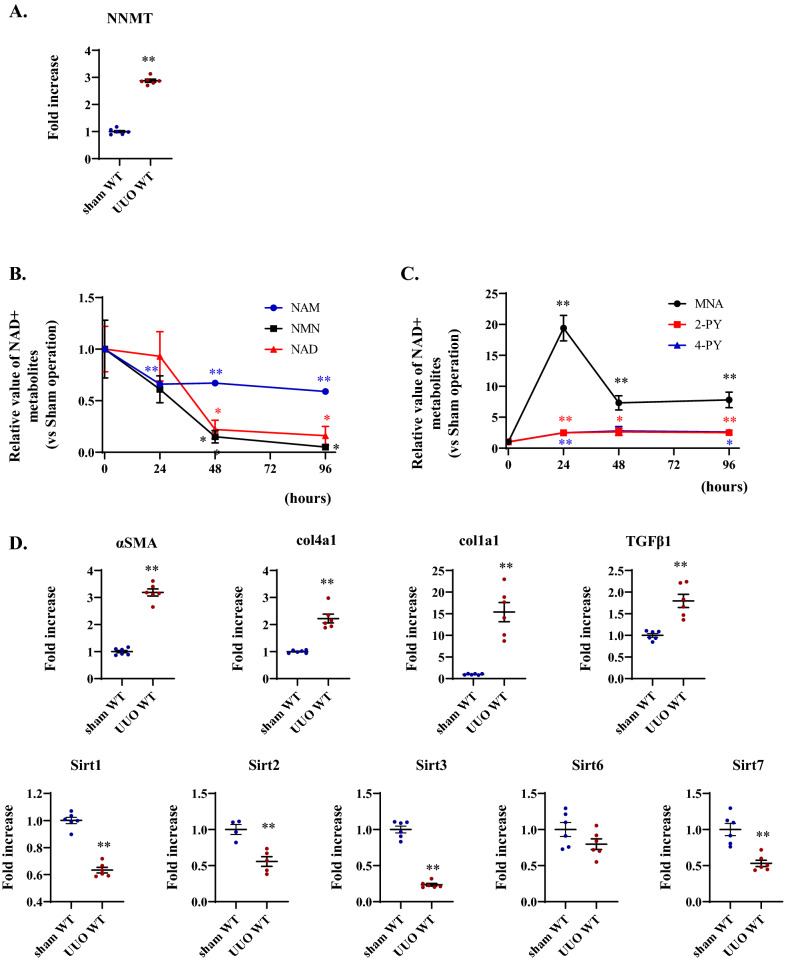
Increased renal NNMT expression and NNMT-mediated NAD + metabolite fluctuations in unilateral ureteral obstruction (UUO) models. (**A**) Renal NNMT expression after four days UUO. The values shown are means ± SEM (*n* = 6 per group). (**B**), (**C**) Time course of renal NAD + metabolite changes after UUO induction. The values shown are means ± SEM (*n* = 3–4 per group). (**D**) mRNA expressions of fibrosis-related genes and sirtuins after four days UUO. The values shown are means ± SEM (*n* = 4–6 per group). UUO, unilateral ureter obstruction; NNMT, nicotinamide N-methyltransferase; NAD + , nicotinamide adenine dinucleotide; NAM, nicotinamide; NMN, nicotinamide mononucleotide; NNO, nicotinamide-N-oxide; MNA, 1-methylnicotinamide; N-Me-2PY, N-methyl-2-pyridone-5-carboxamide; N-Me-4PY, N-methyl-4-pyridone-3-carboxamide; αSMA, α-smooth muscle cell actin; col, collagen; TGF, transforming growth factor. *P < 0.05, **P < 0.01 versus control (sham-operated kidney).

To determine the changes in NAD + metabolites and NNMT expression in renal fibrosis, we used the mouse UUO model. In the UUO model, renal dysfunction is induced concomitantly with renal tissue fibrosis^18,19^. After the induction of the UUO, NNMT mRNA in the kidney was significantly upregulated (Fig. 2A). In addition, upstream metabolites of NNMT (in the NAD + salvage pathway), such as NAM, NMN, and NAD +  (Fig. 1), in renal tissue significantly decreased compared to the sham-operated kidneys (Fig. 2B). In contrast, downstream metabolites of NNMT (the degraded metabolites of NAM), such as MNA, N-Me-2PY, and N-Me-4PY (Fig. 1) accumulated in the obstructed kidneys (Fig. 2C). In the obstructed kidney where NAD + metabolism was dysregulated, the expressions of fibrosis-related genes, such as collagen and *TGFβ1*, increased compared to those in the sham-operated kidney (Fig. 2D). Among several NAD + -dependent sirtuin isoforms, Sirt1, Sirt2, Sirt3, and Sirt7 mRNA expression significantly decreased in the UUO kidneys compared to those in the sham-operated kidneys (Fig. 2D). Therefore, in the renal fibrosis model, renal NNMT expression was elevated, with a concomitant decrease in the upstream metabolites of NNMT and an increase in the downstream metabolites of NNMT.

### NNMT deficiency ameliorates fibrosis in UUO models

**Figure 3:**
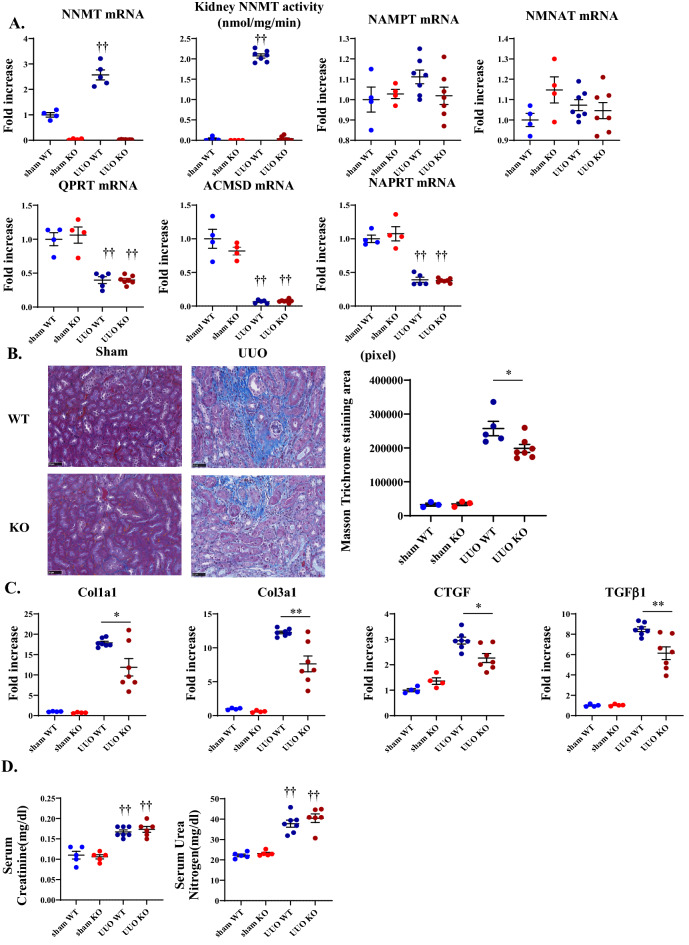
NNMT deficiency ameliorates renal fibrotic changes in the UUO model. (**A**) NNMT mRNA expression and NNMT activity, NAMPT, NMNAT, QPRT, ACMSD, and NAPRT mRNA expression were measured in NNMT-KO mice and WT littermates after UUO induction. The values shown are means ± SEM (*n* = 4–7 per group). (**B**) Renal fibrosis was assessed by Masson-trichrome staining in the kidneys of NNMT-KO mice and WT littermates after UUO induction. The values shown are means ± SEM (*n* = 3–7 per group). (**C**) Fibrosis-related genes were measured in the kidneys of NNMT-KO mice and WT littermates after UUO induction. The data represent means ± SEM (*n* = 4–7 per group). (**D**) Renal function (serum creatinine and urea nitrogen) was measured in NNMT-KO mice and WT littermates after UUO induction. The values shown are means ± SEM (*n* = 4–7 per group). UUO, unilateral ureter obstruction; NNMT, nicotinamide N-methyltransferase; NMNAT, nicotinamide mononucleotide adenyltransferase; NAMPT, nicotinamide phosphoribosyltransferase; QPRT, quinolinate phosphoribosyltransferase; ACMSD, alpha-amino-beta-carboxy-muconate-semialdehyde decarboxylase; NAPRT, nicotinate phosphoribosyltransferase; Col, collagen; *CTGF*, connective tissue growth factor; TGF, transforming growth factor. *P < 0.05, **P < 0.01 versus WT mice. †*P* < 0.05, ††*P* < 0.01 versus the same genotype control mice (sham-operated kidney).

Since the renal NNMT expression was increased in the renal fibrosis model, to determine the causal relationship between NNMT and renal fibrosis, we created an NNMT-deficient (NNMT-KO) mice. These mice were apparently normal, viable, and fertile. We first confirmed that renal NNMT mRNA expression or NNMT activity was not observed under basal conditions in the NNMT-KO mice (Fig. 3A). Four days after UUO induction, renal NNMT expression and activity increased in the WT mice compared to that in the sham WT mice but were not observed in the NNMT-KO mice (Fig. 3A). The expression of the NAD + salvage enzymes, nicotinamide phosphoribosyltransferase (NAMPT) and nicotinamide mononucleotide adenylyltransferase (NMNAT), were similar between NNMT-KO and WT mice (Fig. 3A). The expression levels of alpha-amino-beta-carboxy-muconate-semialdehyde decarboxylase (ACMSD) and quinolinate phosphoribosyltransferase (QPRT), which constitute the de novo pathway, and nicotinate phosphoribosyltransferase (NAPRT), which is the rate-limiting enzyme in the Preiss–Handler pathway, were significantly decreased in the WT-UUO mice as compared to the WT-sham. However, there were no significant differences between WT-UUO and KO-UUO (Fig. 3A).

Thereafter, we examined the degree of fibrosis in NNMT-KO mice and WT mice in the UUO model. Fibrosis was evaluated by blue areas of Masson-trichrome staining, which significantly decreased in the NNMT-KO mice compared to the WT mice after UUO induction (Fig. 3B). The expression of fibrosis and fibrosis-related genes, such as *col1a1, col3a1, CTGF,* and *TGFβ1*, decreased significantly in NNMT-KO mice compared to that in the WT mice (Fig. 3C). Moreover, we examined the degree of fibrosis in NNMT transgenic (NNMT-Tg) mice^15^ and WT mice four days after UUO induction. In contrast to the NNMT-WT mice, NNMT-Tg mice showed increased tubular interstitial fibrosis (Supplementary Fig. 1A) and fibrosis-related genes (Supplementary Fig. 1B) after UUO induction. Therefore, studies on the NNMT-KO (Fig. 3) and NNMT-Tg mice (Supplementary Fig. 1) demonstrated that NNMT promotes renal fibrosis i*n vivo*. Serum creatinine and urea nitrogen levels were significantly elevated in UUO compared to their values in the sham-operated mice, suggesting reduced renal function. However, no significant differences in renal function were observed in the NNMT-KO/Tg mice UUO model (Fig. 3D, Supplementary Fig. 1C).

### NNMT deficiency increases SAM/SAH ratio and epigenetically reduces *CTGF* expression

**Figure 4:**
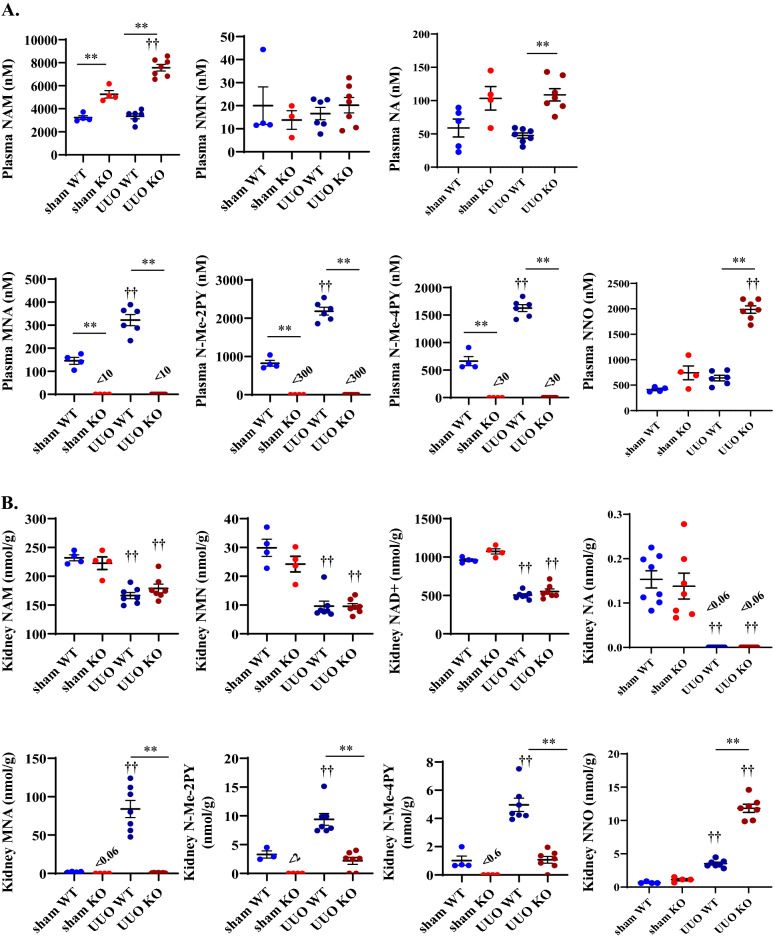

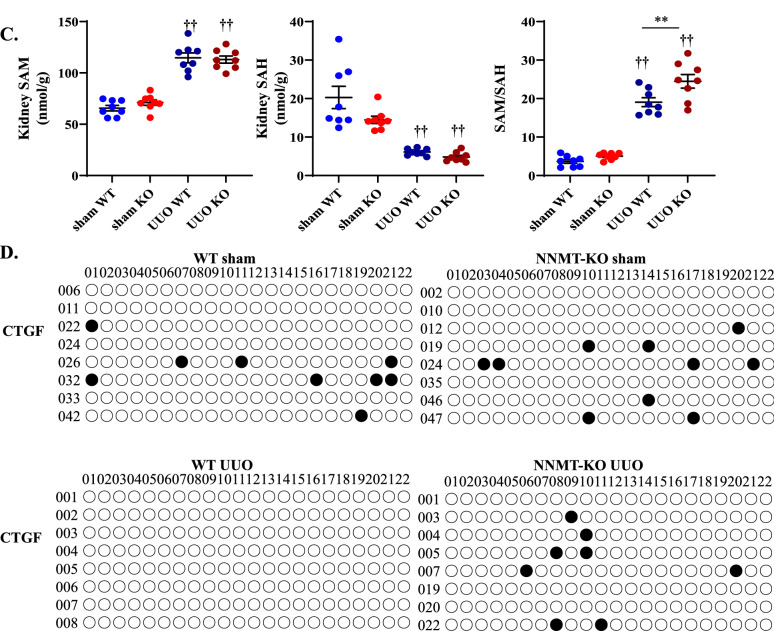
NNMT deficiency decreases renal N-Me-2PY and N-Me-4PY and increases SAM/SAH ratio in UUO, thereby increasing the methylation capacity of *CTGF*. Plasma NAD + metabolites (**A**) and renal NAD + metabolites (**B**) were measured in NNMT-KO mice and WT littermates after UUO induction. The values shown are means ± SEM (*n* = 4–8 per group). (**C**) SAM levels, SAH levels, and SAM/SAH ratios in the kidneys of NNMT-KO mice and WT littermates after UUO induction. The data represent means ± SEM (*n* = 8 per group). (**D**) Bisulfite sequencing of *CTGF CpG* islands. Each circle indicates a CpG site in the sequence, and each line of circles represents a single cloned allele. Open circles, unmethylated CpG sites; closed circles, methylated CpG sites. UUO, unilateral ureter obstruction; NNMT, nicotinamide N-methyltransferase; NAM, nicotinamide; NMN, nicotinamide mononucleotide; NAD + , nicotinamide adenine dinucleotide; MNA, 1-methylnicotinamide; N-Me-2PY, N-methyl-2-pyridone-5-carboxamide; N-Me-4PY, N-methyl-4-pyridone-3-carboxamide; NA, nicotinic acid; NNO, nicotinamide-N-oxide; SAM, S-adenosylmethionine; SAH, S-adenosylhomocysteine; CTGF, connective tissue growth factor. *P < 0.05, **P < 0.01 versus WT mice. †*P* < 0.05, ††*P* < 0.01 versus the same genotype control mice (sham-operated kidney).

To elucidate the mechanism of fibrosis improvement, we examined the NAD + metabolites in plasma and renal tissues and methionine metabolites, such as SAM and SAH, in renal tissues. Plasma NAM concentration was elevated in NNMT-KO mice, even in the sham-operated mice (Fig. 4A), which is consistent with previous studies on the NNMT-KO mouse model^20^. Moreover, after UUO induction, plasma NAM concentration further increased in the NNMT-KO mice. (Fig. 4A). Plasma MNA, N-Me-2PY, and N-Me-4PY were significantly decreased in the NNMT-KO mice regardless of UUO induction, suggesting increased plasma levels of N-Me-2PY and N-Me-4PY in the UUO model was not correlated with a decrease in clearance, although influenced by NNMT upregulation (Fig. 4A). Thereafter, we measured the NAD + metabolite concentration in renal tissues. In the UUO model, NAD + concentration in the kidney decreased compared to that in the sham-operated mice (Fig. 4B). NNMT deficiency did not affect renal NAD + levels at this time point; however, MNA, N-Me-2PY, and N-Me-4PY concentrations in the kidney significantly decreased in the NNMT-KO mice (Fig. 4B). In addition, nicotinamide-N-oxide (NNO) was increased in UUO mice compared to that in the sham-operated mice. A further increase in NNO was observed in the NNMT-KO mice. The fact that renal NAM decreased while MNA and NNO increased in the WT-UUO compared to their respective values in the WT-sham indicated that the NAM excretion pathway is augmented in the UUO model (Fig. 4B). The increase was more remarkable for MNA than NNO in WT-UUO, suggesting that NAM is primarily metabolized to MNA by NNMT in UUO. In addition, kidney NA was markedly decreased in UUO compared to that in sham-operated mice (Fig. 4B).

Thereafter, we examined whether NNMT suppression alters SAM/SAH ratio. Increased NNMT activity reduced SAM and increased SAH levels (Fig. 1), thereby reducing methylation capacity by decreasing the SAM/SAH ratio^13^. Compared to that in the sham-operated mice, the renal SAM showed a significant increase in the UUO mice, while the renal SAH showed a significant decrease, effectively increasing the renal SAM/SAH ratio significantly in the UUO mice. Additionally, the renal SAM/SAH was further elevated in the NNMT-KO-UUO model compared to that in the WT-UUO (Fig. 4C). Therefore, we examined the DNA methylation at sites that regulate *CTGF* expression; importantly, the methylation status around the CpG island partially associated with *CTGF* genes is known to regulate *CTGF* expression^15,21^. *CTGF* gene bisulfite sequencing demonstrated significantly increased DNA methylation in the obstructed kidney of NNMT-KO mice (Fig. 4D). Therefore, one of the mechanisms of fibrosis amelioration following NNMT suppression was increased *CTGF* methylation by upregulating the SAM/SAH ratio, possibly through epigenetic changes.

In addition, to examine the effect of NNMT on oxidative stress, we performed 4-HNE (4-hydroxy-2-nonenal) staining. An increase in the stained area was observed in the UUO mice compared to that in the sham-operated mice. However, there was no significant change in the NNMT-KO-UUO mice (Supplementary Fig. 2A). Thereafter, we measured NAD + /NADH and NADP/NADPH. Total NAD (NAD + plus NADH) was decreased in the UUO model. NNMT deficiency did not affect NAD + /NADH and NADP/NADPH levels (Supplementary Fig. 2B).

We also measured NNMT expression and NAD + metabolites in the adenine-induced CKD model. In the adenine model, renal NNMT expression was significantly upregulated (Supplementary Fig. 3A). In addition, kidney and plasma NAD + metabolites generally showed a similar trend as in the UUO model. NAD + in kidney tissue was significantly decreased in the adenine model, but there were no significant differences between WT and KO (Supplementary Figs. 3B,C).

### NNMT deficiency reduces renal inflammation by increasing renal NAD + and Sirt1, decreasing NF-κB acetylation

**Figure 5:**
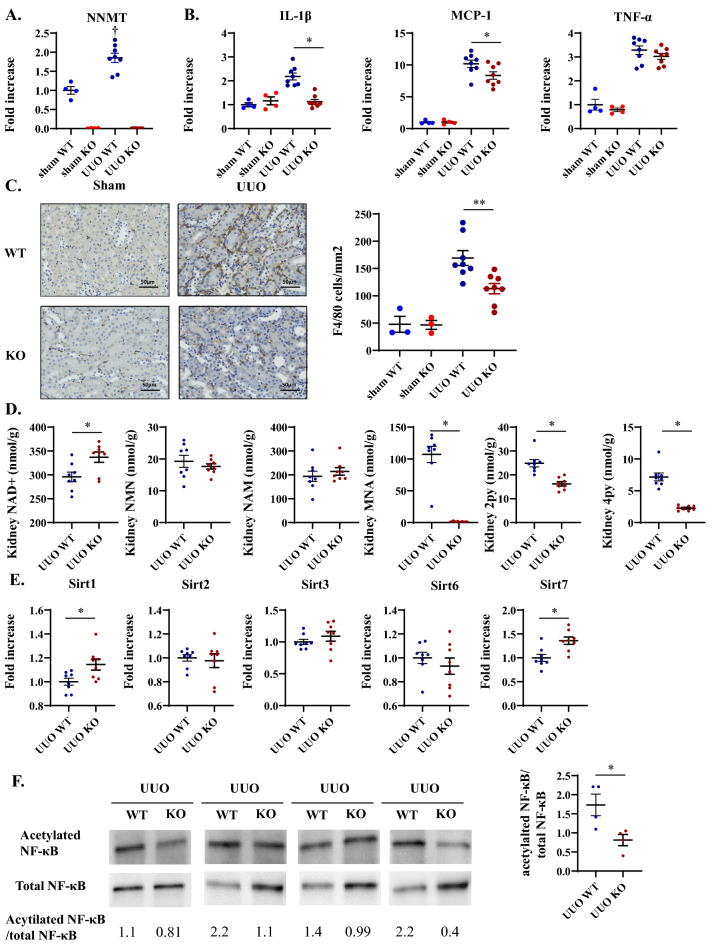
NNMT deficiency ameliorates renal inflammation in the UUO model. Renal NNMT mRNA expression (**A**), inflammation-related genes (**B**), renal NAD + metabolites (**D**), and mRNA expression of sirtuins (**E**) were measured in the kidneys of NNMT-KO mice and WT littermates two days after UUO induction. The data represent means ± SEM (*n* = 4–8 per group). (**C**) IHC staining and quantitative analysis for F4/80. The data represent means ± SEM (*n* = 3–8 per group). (**F**) The acetylation status of NF-κB in the kidney after UUO induction (*n* = 4 per group). Full-length blots/gels are presented in Supplementary Fig. 7. UUO, unilateral ureter obstruction; NNMT, nicotinamide N-methyltransferase; Il-1β, interleukin 1-beta; MCP-1, monocyte chemotactic protein-1; TNF-α, tumor necrosis factor-alpha; NAM, nicotinamide; NMN, nicotinamide mononucleotide; NAD + , nicotinamide adenine dinucleotide; MNA, 1-methylnicotinamide; N-Me-2PY, N-methyl-2-pyridone-5-carboxamide; N-Me-4PY, N-methyl-4-pyridone-3-carboxamide; NF-κB, nuclear factor-kappa B. *P < 0.05, **P < 0.01 versus WT mice; †*P* < 0.05, ††*P* < 0.01 versus the same genotype control mice (sham-operated kidney).

To further elucidate the mechanism underlining the amelioration of renal fibrosis, we examined the inflammation in the kidney at an earlier stage of UUO. NNMT expression and inflammation-related genes, such as *IL-1β, MCP-1,* and *TNF-α*, were elevated in UUO mice compared to that in the sham-operated mice (Fig. 5A,B)^22,23^. Moreover*, IL-1β* and *MCP-1* mRNA levels were significantly decreased in NNMT-KO mice compared to the WT mice two days after UUO induction (Fig. 5B). Thereafter, we examined the invasion of macrophages. In NNMT-KO mice, the number of F4/80 positive cells was decreased compared to that in the WT mice (Fig. 5C). Renal NAD + concentration was reduced as early as two days after the induction of UUO (Fig. 2B), and NNMT deficiency significantly increased renal NAD + levels at this time point (Fig. 5D). With the increased NAD + level, Sirt1 and Sirt7 expression were elevated in NNMT-KO mice (Fig. 5E). Sirt1 represses tissue inflammation through NF-κB^24,25^. In NNMT-KO mice, the acetylation of NF-κB was decreased compared to that in the WT mice (Fig. 5F), resulting in reduced renal inflammation. The effect of Sirt7 on inflammation is controversial^26,27^. These observations suggest that the improved renal inflammation through the Sirt1-mediated deacetylation of NF-κB is another mechanism contributing to the amelioration of fibrosis following NNMT suppression.

### The profile of NAD + metabolites varied based on CKD progression

Since NNMT and NAD + metabolites were important in renal fibrosis in the mouse model, we decided to determine the significance of NAD + metabolism in patients with CKD. First, we analyzed serum and urinary NAD + metabolite levels. Supplementary Table 1 summarizes the characteristics of the study participants grouped by CKD stages. This study included 139 patients from 57 to 78 years old (median age: 69 years), which consisted of 85 men (61.0%), 110 participants with hypertension (79%), 38 with diabetes (27%), and 73 with hyperuricemia (53%). Ages were significantly higher concomitant with CKD groups, and stage 1 participants were younger than other stage participants. As expected, the prevalence of hypertension, diabetes, and hyperuricemia was higher in patients with advanced CKD stages (*p* < 0.01 by the Cochran–Armitage trend test). Urinary protein and albumin were also significantly higher in participants with advanced CKD stages (*p* < 0.01). We observed no significant differences among CKD groups regarding BMI, HbA1c, and gender.

**Figure 6:**
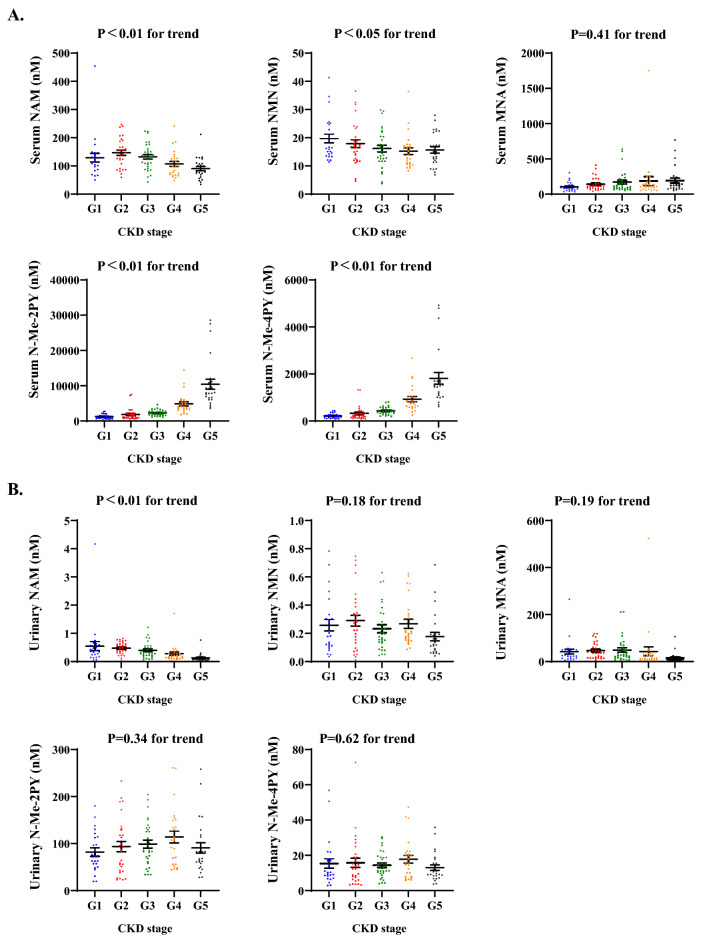
Serum levels of upstream metabolites of NNMT, NAM, and NMN, are reduced, while downstream metabolites of NNMT, N-Me-2PY, and N-Me-4PY are elevated with CKD progression. Scatter plots demonstrating serum (**A**) and urinary (**B**) NAD + metabolites across CKD clinical stages. The data represent means ± SEM. NAM, nicotinamide; NMN, nicotinamide mononucleotide; MNA, 1-methylnicotinamide; N-Me-2PY, N-methyl-2-pyridone-5-carboxamide; N-Me-4PY, N-methyl-4-pyridone-3-carboxamide.

The relationship between serum NAD + metabolites and CKD stages are shown in Fig. 6A. In humans, serum NAM concentration was about tenfold higher than NMN concentration, consistent with the previous reports^8^. A previous study reported that the serum NAM concentration increased in uremic patients^11^; however, in the present study, serum NAM concentrations were significantly lower (*p* < 0.01). Moreover, the serum NMN levels gradually decreased with increasing CKD stages (*p* < 0.05). On the other hand, the serum N-Me-2PY and N-Me-4PY levels were significantly higher (*p* < 0.01) (Fig. 6A). We also examined the correlation between serum NAD + metabolites and eGFR (Supplementary Fig. 4). NMN and NAM, precursors of NAD + synthesis, were significantly decreased as renal function declined (NMN: coefficient, 0.050; 95% CI, 0.018 to 0.082; *p* < 0.01, NAM: coefficient, 0.43; 95% CI, 0.17 to 0.69; *p* < 0.01). Conversely, serum N-Me-2PY and N-Me-4PY levels were higher as renal function deteriorated (N-Me-2PY: coefficient, − 75.90; 95% CI, − 95.03–− 56.77; *p* < 0.01, N-Me-4PY: coefficient, − 13.45; 95% CI, − 16.78–− 10.13; *p* < 0.01). We next analyzed urinary NAD + metabolites (Fig. 6B). We found that only urinary NAM concentration was significantly lower with advancing CKD stages. The association of urinary NAM and eGFR was also examined. Urinary NAM was significantly lower as renal function declined (Supplementary Fig. 5).

The associations between NAD + metabolite concentration and various factors (eGFR, HbA1c, age, and BMI), which are expected to influence serum NAD + metabolite levels, were examined using multiple linear regression analyses. Not only eGFR but also age was negatively associated with serum N-Me-2PY and N-Me-4PY. The level of eGFR was the only explanatory variable for serum and urinary NAM (Supplementary Table 2).

Therefore, in CKD, the variations in serum NAD + metabolites were possibly influenced by NNMT since the upstream metabolites of NNMT, such as NAM and NMN (NAD + precursors), were declined, while the downstream metabolites of NNMT, such as N-Me-2PY and N-Me-4PY (final NAD + metabolites), were elevated with CKD progression. When combined with the mouse results (Fig. 4A), the increase in serum levels of N-Me-2PY and N-Me-4PY suggests an increase in NNMT expression in patients with CKD.

### NNMT expression is upregulated in the renal fibrosis area in human CKD

**Figure 7:**
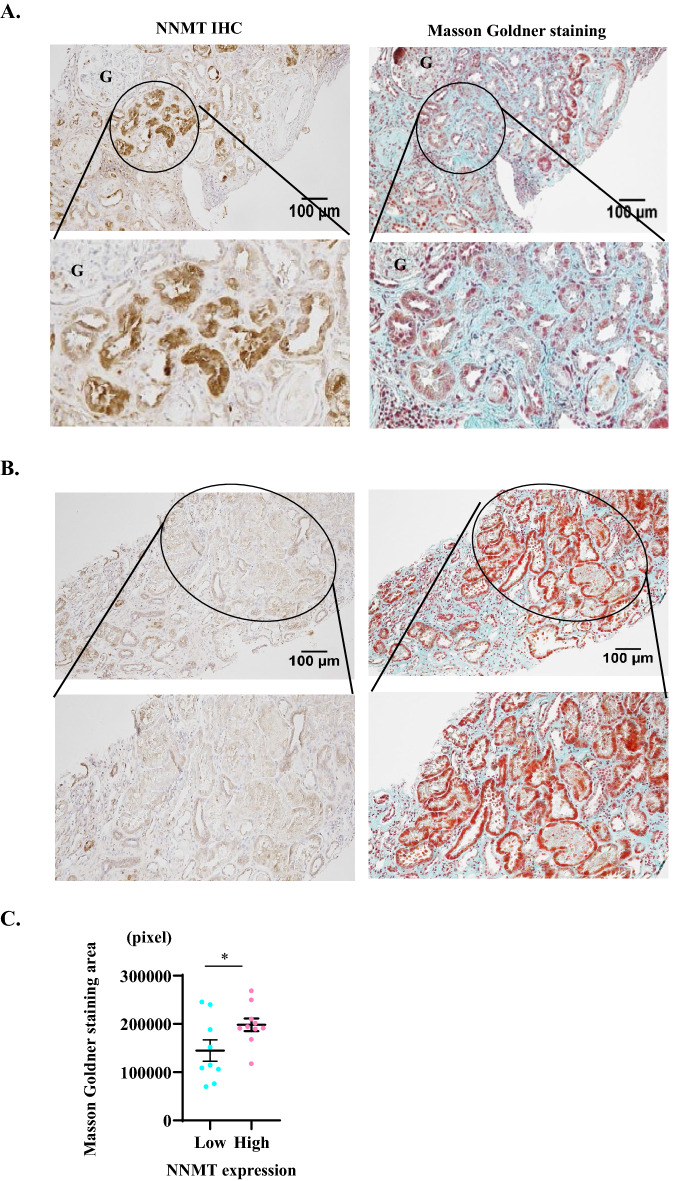
NNMT expression correlates with renal fibrosis in human kidney tissues. Representative image including a region with high NNMT expression (**A**) and with low NNMT expression (**B**) in human fibrotic kidney tissue and enlarged view of the circled area (left panel). Masson Goldner staining in the same area and enlarged view of the circled area (right panel). Bars represent 100 µm. (**C**) The high NNMT expression group (*n* = 10) is significantly associated with renal fibrosis measured by the blue area of Masson Goldner staining, compared to the low NNMT expression group (*n* = 9). NNMT, nicotinamide N-methyltransferase; IHC; immunohistochemistry. *P < 0.05 versus low NNMT expression group.

Although the alterations in serum NAD + metabolites in patients with CKD suggested that NNMT expression is also increased in CKD, the NNMT expression level in the kidney and the site of NNMT expression remain unclear. To investigate the expression of NNMT and its relationship with renal fibrosis in human kidney tissue, we used human renal biopsy samples. Clinical characteristics of the patients are shown in Supplementary Table 3. Immunohistochemistry revealed that NNMT was present in atrophic tubular cells around fibrotic lesions, as demonstrated by Masson Goldner staining (Fig. 7A,B). Figure 7A is a representative image of high NNMT expression in the fibrotic region; moreover, we found progressive fibrosis on Masson-trichrome staining. On the contrary, Fig. 7B shows low NNMT expression, suggesting mild renal fibrosis and preservation of renal tubules using Masson-trichrome staining. To investigate the relationship between NNMT expression and renal fibrosis, we compared the area of fibrosis between the high and low NNMT expression groups (high group, *n* = 10; low group, *n* = 9) and found that high NNMT expression was significantly associated with the high degree of renal fibrosis (Fig. 7C). These results suggest that elevated NNMT expression may also be associated with renal fibrosis in human CKD.

## Discussion

**Figure 8:**
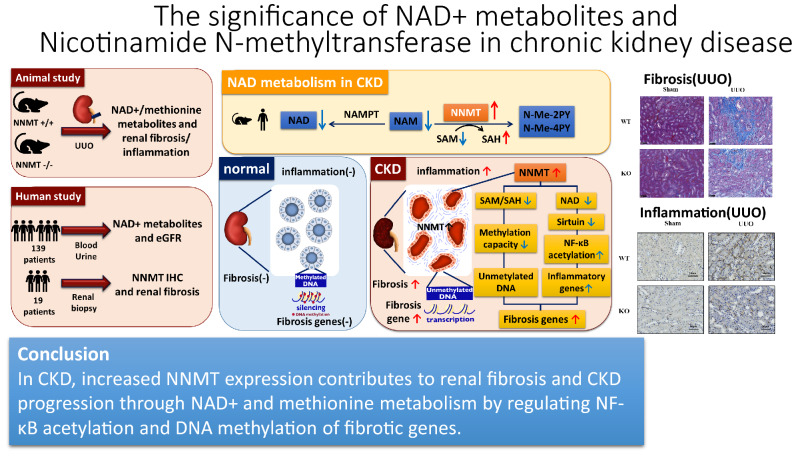
Graphical abstract of this study. In CKD, increased NNMT expression contributes to renal fibrosis and CKD progression through NAD + and methionine metabolism by regulating NF-κB acetylation and DNA methylation of fibrotic genes. UUO, unilateral ureter obstruction; NAM, nicotinamide; NAD + , nicotinamide adenine dinucleotide; NAMPT, nicotinamide phosphoribosyltransferase; NNMT, nicotinamide N-methyltransferase; N-Me-2PY, N-methyl-2-pyridone-5-carboxamide; N-Me-4PY, N-methyl-4-pyridone-3-carboxamide; SAM, S-adenosylmethionine; SAH, S-adenosylhomocysteine; NF-κB, nuclear factor-kappa B.

In this study, we showed an increase in renal NNMT expression and NNMT-mediated changes in renal NAD + metabolites in a mouse model of renal fibrosis; renal NAD + salvage pathway metabolites, such as NAM, NMN, and NAD + , were diminished, while degradation products of NAM, such as MNA, N-Me-2PY, and N-Me-4PY, were increased in renal fibrosis. These trends in NNMT expression and NAD + metabolite levels were similar to those observed in another CKD model, the adenine model. These results indicated that the NNMT induction presumably dictates the signature of renal NAD + metabolites in kidney fibrosis. Moreover, using NNMT-KO mice, we demonstrated that NNMT deficiency ameliorated UUO-induced renal fibrotic changes by increasing methylation of the profibrotic *CTGF* gene and the attenuation of renal inflammation. Besides, we profiled kidney samples from 139 patients with CKD and found that serum concentrations of N-Me-2PY and N-Me-4PY tended to elevate with CKD progression. Conversely, serum concentrations of NAD + salvage pathway metabolites, including NAM and NMN, were reduced with the advancement of CKD stages. This pattern of NAD + metabolite was also suggestive of increased NNMT expression in the context of human CKD. Indeed, upregulation of NNMT was observed alongside the fibrotic areas in human kidney samples. Remarkably, multiple regression analyses, adjusted for clinical variables, demonstrated that eGFR was independently associated with serum levels of NAD + metabolites, such as NAM, N-Me-2PY, and N-Me-4PY. These suggested that the NNMT upregulation with CKD progression is most likely involved in renal fibrotic changes (Fig. 8).

NAD + levels decrease with age, and the reduction of NAD + contributes to the progression of age-associated diseases^28^. In this study, renal NAD + and precursors for NAD + synthesis (NAM and NMN) decreased in the mouse CKD model (Fig. 2B). Since NAD + is an essential cofactor for redox reaction pathways, such as glycolysis and fatty acid oxidation, decreased fatty acid oxidation in tubular cells accelerates renal injury^29^. NAD + is also a substrate for NAD + -consuming enzymes, such as sirtuins, poly ADP ribose polymerase, and CD38. Sirt1 and Sirt3 exert reno-protective effects through several mechanisms, such as promoting mitochondrial function, reducing oxidative stress, and blocking TGFβ signaling^4,30^. Recently, we reported that decreased Sirt6 activity in the proximal tubule decreases renal fibrosis^31^. In addition, NMN and NAM administration has attracted attention as a NAD-boosting therapy in CKD. We have previously reported that NMN administration improves albuminuria in diabetic nephropathy^32^. Additionally, NAM administration improves renal fibrosis^33,34^. Taken together, decreased NAD + and precursors for NAD + synthesis in CKD could contribute to CKD progression.

NAD + and its precursors are regulated in tissues as follows: (1) the de novo pathway that synthesizes NAD + from tryptophan, (2) NAD + synthesis from nicotinic acid through the Preiss–Handler pathway, (3) the salvage pathway that synthesizes NAD + from NAM via NAMPT, (4) via NAD + -consuming enzymes, and (5) the NAM excretion pathway by NNMT (Fig. 1). In acute kidney injury, a combination of accelerated NAD + consumption and reduced NAD + biosynthesis is a significant factor for reduced renal tissue NAD + levels^35^. In CKD, the de novo pathway, including altered tryptophan metabolism, is involved in decreased NAD + levels^36,37^. However, data regarding the role of NNMT in CKD and renal fibrosis is lacking. This study showed that NNMT expression and activity increased, and the NAM excretion pathway by NNMT also increased. Moreover, the change in the expression of enzymes associated with the de novo pathway and Preiss–Handler pathway suggested that NAD + synthesis by those pathways was reduced in the renal injury model (Fig. 3A). Besides, the results from mice experiments indicated that the increase in plasma N-Me-2PY and N-Me-4PY levels during renal failure did not correlate with a decrease in clearance; rather, it was influenced by NNMT (Fig. 4A). This suggests that the increase in plasma N-Me-2PY and N-Me-4PY levels was correlated with increased NNMT expression. Therefore, since serum N-Me-2PY and N-Me-4PY increased with CKD progression in humans, NNMT expression may be increased. Moreover, NNMT depletion improved renal fibrosis (Fig. 3B,C), and renal NAD + increased at an early stage of UUO (Fig. 5D). Previous studies have reported that NNMT regulates NAD + tissue levels and that NNMT knockdown by antisense oligo or pharmacological inhibition increases NAD + content^14,38^. In the present study, genetic NNMT knockout increased renal NAD + level on Day 2 after UUO induction; however, it did not affect NAD + content in renal tissues on Day 4 after UUO induction (Figs. 4B and 5D). It is probably because the activity of NNMT peaks at early time points in UUO models, as indicated by the fact that renal MNA concentration increased as early as on Day 1 and decreased over time (Fig. 2C). In addition, it is reported that NNMT knockout increases NAD + synthesis by increasing the availability of its precursor NMN (Fig. 1)^14^. However, in this study, neither renal NAD + nor NMN was elevated in KO-UUO. Instead, NNO was significantly elevated in KO-UUO at Day 4 (Fig, 4B), indicating that NAM is mainly metabolized to NNO in NNMT-KO mice. The enzyme CYP2E1 metabolizes NAM to NNO^39^. We speculate that NAM excretion could be enhanced by CYP2E1 in the absence of NNMT, resulting in the unexpected lack of increase in NAD + by NNMT-KO in UUO condition at Day 4. In addition, renal NA was markedly decreased in UUO; and NAD + synthesis from the Preiss–Handler pathway was predicted to decrease in the kidney. In addition to N-Me-2PY and N-Me-4PY, NAD + degradation also produces the ribosylated form of the pyridone carboxamides, such as 2PYR, 4PYR, and 6PYR (PYR: pyridine riboside) (Fig. 1). Unlike N-Me-2PY and N-Me-4PY, PYR is not metabolized via MNA but rather from NAD + ^40^, and PYR is reported to be elevated in renal failure^40^. Therefore, it is expected that suppression of NNMT does not decrease PYR in UUO. In addition, it has been reported that PYR decreases NAD + ^41^. This may be one of the reasons why NAD + was not significantly elevated in the NNMT-KO condition at UUO Day 4.

To elucidate the mechanism of improved fibrosis by NNMT suppression, we first examined NAD + and methionine metabolites in renal tissues (Fig. 4B,C) because NNMT links NAD + and methionine metabolism (Fig. 1). There was no significant elevation in NAD + in NNMT-KO mice four days after UUO induction (Fig. 4B). Interestingly, an increase in the SAM/SAH ratio was observed. On the other hand, in the sham-operated mice, DNA methylation of *CTGF* was observed (Fig. 4D) even with a low SAM/SAH ratio (Fig. 4C), indicating that SAM/SAH alone does not regulate the gene methylation status. SAM/SAH and DNA methyltransferase (DNMT) have been reported to regulate DNA methylation in the DNA methylation and ten–eleven translocation (TET) enzyme family and activation-induced deaminase (AID)/APOBEC for DNA demethylation^42^. The methylation status around the *CTGF CpG* island correlated inversely with the expression, and this target region for methylation showed promoter activity^43^. Bisulfite sequencing of *CTGF* DNA from the WT-UUO animals revealed a decrease in methylation in the search range compared to that in the WT-sham. In addition, the methylation status was restored to the same extent in KO-UUO. However, *CTGF* expression was higher in KO-UUO than in WT/KO-sham. This is probably because the *CTGF* expression is not only regulated by DNA methylation but also by histone acetylation and *Smad*, TGF-β-mediated-transcription factor, which is augmented in the UUO model^44^. NNMT activation produces MNA coupled with reduced methylation capacity through decreasing SAM/SAH ratio^13^. Hypomethylation of various profibrotic genes, such as *CTGF,* collagen type I alpha 1 chain (*col1a1*), and plasminogen activator inhibitor-1, could induce epigenetic changes and renal fibrosis^45–48^. Previously, we demonstrated that NNMT activation promotes hypomethylation of the *CTGF* gene promoter, increases *CTGF* mRNA expression, and eventually promotes collagen deposition in the liver^15^. This study revealed that renal fibrosis occurs by the same mechanism of epigenetics as in the liver.

To further determine the mechanism responsible for ameliorating renal fibrosis, we examined renal inflammation at an earlier stage after UUO induction. We found that NNMT deficiency represses tissue inflammation through increased renal NAD + levels, which in turn increased sirtuin expressions (Fig. 5E) and decreased acetylation of NF-κB (Fig. 5F). Moreover, kidney fibrosis is preceded by and closely related to chronic interstitial inflammation^49,50^. Consequently, reducing renal inflammation at an earlier stage in NNMT-KO mice (Fig. 5) could ameliorate renal fibrosis (Fig. 3) after UUO induction. Moreover, since the relationship between NNMT and oxidative stress has not been consistently reported^51,52^, we performed 4-HNE staining to investigate it further. Oxidative stress was significantly increased in UUO compared to that in sham-operated mice. However, no significant change was observed in NNMT-KO on UUO induction (Supplementary Fig. 2A). In other words, the present UUO model did not suggest that NNMT-KO improved oxidative stress, and the NADP/NADPH results (Supplementary Fig. 2B) also indicated that oxidative stress was unlikely to be the mechanism for the aggravation of renal fibrosis in NNMT-KO. Therefore, this study demonstrated that NNMT is increased upon renal injury and exacerbates renal fibrosis through NAD + and methionine metabolism. In other words, alleviation of renal fibrosis due to NNMT deficiency might be mediated through the elevation of renal NAD + and SAM/SAH ratio. Administrating NAD + precursors and methionine to augment this effect might provide a more robust explanation of the underlying mechanism. As for NAD + precursors, it has been reported that NAM administration improves renal fibrosis^33,34^. On the other hand, methionine has been reported to increase renal tubular damage^53^, and methionine administration does not always increase SAM/SAH^54^. Further studies are needed to elucidate the effect of NAD + precursors or methionine. In addition, NNMT also participates in several cellular processes, such as reactive oxygen species generation, the NAD + /NADH ratio, and polyamine flux^14,55^, which could contribute to tissue fibrotic changes. Alternatively, NNMT products, such as MNA and N-Me-2PY, have biological effects^11,56^. For example, MNA improves lipotoxicity-induced oxidative stress in proximal tubular cells^57^, and N-Me-2PY may decrease kidney function by inhibiting poly ADP ribose polymerase activity^17^. Thus, NNMT products might contribute to the progression of renal injury. In addition to the effects of NNMT and its products, there are also reports stating that increased NNMT expression deteriorates obesity^14^ and increases insulin sensitivity^20^. We also reported that increased NNMT expression exacerbates liver fibrosis^15^. On the other hand, it has been reported that NNMT expression is regulated by inflammation. In vitro experiments have shown that direct stimulation of human skeletal myoblasts with IL-6, TNFα, and TGFβ increases NNMT expression, which may be a compensatory response to injury, including oxidative stress. It is challenging to determine whether NNMT is elevated as a result of injury or whether elevated NNMT causes the injury. However, this study clearly showed that NNMT deficiency and overexpression resulted in lower and higher renal fibrosis respectively (Fig. 3 and Supplementary Fig. 1), suggesting that NNMT contributed to the worsening of inflammation and fibrosis. Further investigation is needed to elucidate the regulation of NNMT expression and its effects, primarily on renal fibrosis.

In conclusion, we demonstrated that NAD + metabolism is altered in human and mouse models of CKD. Increased NNMT expression induces NAD + and methionine metabolism disturbance, thereby aggravating renal fibrosis through the regulation of DNA methylation of fibrotic genes and acetylation of NF-κB in the mouse model, and NNMT elevation may also be associated with renal fibrosis in human CKD. Therefore, increased NNMT expression in the kidney and the associated changes in NAD + metabolism most likely contribute to the progression of renal fibrosis.

## Methods

### Animal study

All animal experiment protocols were approved by the Institutional Animal Care and Use Committee of Keio University (Tokyo, Japan) (Approval No. 15010), and all experiments were performed following relevant guidelines and regulations and in compliance with the ARRIVE guidelines. The role of NAD + metabolites and NNMT on renal fibrosis was explored by using the mouse unilateral ureteral obstruction (UUO) model. In the UUO model, unilateral urethral ligation was performed in age-matched mice (C57BL/6, male, 8–16 weeks). Under anesthesia, the left ureter was exposed via a flank incision and ligated with surgical sutures at two places. Thereafter, the peritoneum and skin were stitched. Mice were dissected at the indicated time points after UUO treatment. Control animals were subjected to sham operations^58^. NNMT transgenic (Tg) mice were generated previously^15^. In the adenine model, age-matched mice (C57BL/6, male, 8 weeks) were randomly sorted into control and adenine-induced CKD groups. Control mice were fed a standard diet (CE-2), while the CKD group was fed a CE-2 diet supplemented with 0.2% adenine (Wako, Osaka, Japan) for 6 weeks. All surgeries were performed after intraperitoneal injection of 0.3 mg/kg medetomidine, 4.0 mg/kg midazolam, and 5.0 mg/kg butorphanol^59^, and all efforts were made to minimize animal suffering^15^.

### Development of NNMT knockout (KO) mice

We generated an NNMT-KO mouse line according as reported previously, with minor modifications^60^. CompoZr Knockout Zinc Finger Nucleases (ZFN) Kits designed to target exon 2 of the mouse *Nnmt* gene were purchased from Sigma-Aldrich (St. Louis, MO, USA). ZFN mRNA (10 ng/μL) was injected into single-cell C57BL/6 embryos. The embryos were implanted into pseudopregnant ICR female mice (CLEA, Tokyo, Japan). To detect insertion-deletion mutations in founders, the heteroduplex mobility assay (HMA) was performed^61^ using DNA extracted from ear clips of the pups and the following primers: HMA forward primer 5′-TCCCTTTTGATGCCTGATTC-3′; HMA reverse primer 5′-GCATTTTTCATCTGGATCTTAGTGGTAG-3′. HMA-positive PCR products were analyzed by direct sequencing to identify the frameshift mutation (10-base deletion in exon 2, 177–186). After confirmation of germ-line transmission, homozygous knockout mice were generated by inbreeding heterozygous mice. Genotyping PCR was performed with DNA extracted from tails or ear punches and the following primers: wild type forward primer 5′-GACTGGTCCCCAGTGGTCACCTATG-3′; KO forward primer 5′- CCTTTGACTGGTCCCCAGTGTGT-3′, universal reverse primer 5′- GGATGACTGGTAACCAAATAGTACTCTGGTGG-3′.

### Real-time PCR

Total RNA was extracted from mouse kidney tissues using TRIzol reagent. Equal amounts (1 µg) of total RNA from each sample were converted to cDNA using PrimeScript RT Reagent Kits with gDNA Eraser (TaKaRa, Ohtsu, Japan) in 20 µL reactions. Real-time PCR was performed using an ABI Step One Plus Real-Time PCR system (PE Applied Biosystems, Tokyo, Japan), with the amplification program set at 95 °C for 3 min, followed by 40 cycles of 95 °C for 10 s, 62 °C for 10 s, and 72 °C for 10 s. Primer details are available upon request.

### Determination of NNMT activity

We used a previously reported fluorometric method to measure NNMT activity in the murine kidney^62^. This method is based on the enzymatic reaction of NNMT at pH 8.5 using 4-methyl nicotinamide (4-MNA) as the substrate in the presence of cofactor, S-adenosyl-L-methionine (SAM), followed by fluorometric determination of the product, 1,4-dimethylnicotinamide reacted with 4-methoxybenzaldehyde. Kidney tissue was excised, frozen on dry ice, and stored at − 80 °C. The tissue was homogenized 1:9 (w/v) in cold PBS buffer (pH 7.4) with a tissue homogenizer. The homogenate was centrifuged at 12,000 × *g* for 20 min at 4 °C. The supernatant was used immediately to assay enzymatic activity. Protein concentration was determined using BSA standards. The reaction mixture consisted of 1 μL dimethylsulfoxide, 6.25 μL 2 mM dithiothreitol, 5 μL 1 M Tris–HCl buffer (pH 8.5), 25 μL 16 mM 4-MNA, 25 μL 0.4 mM SAM in 0.1 mM sulfuric acid, 12.75 μL distilled water, and 25 μL enzyme preparation. The mixture was incubated at 37 °C for 30 min. Substrate N-methylation proceeded linearly during the incubation period. The enzyme reaction was terminated by boiling at 37 °C for 4 min. The same procedure was used to prepare the blank sample. These reaction mixtures were centrifuged at 13,000 × *g* for 5 min at room temperature. A 27.3-μL aliquot of the supernatant was poured into a 1.5-ml stopper test tube and mixed with 273 μL 0.02 M 4-methoxybenzaldehyde in 35% (v/v) aqueous 2-methoxyethanol and 27.3 μL 0.5 M aqueous sodium hydroxide. The tube was heated in an incubator at 100 °C for 15 min. After cooling, the reactants were centrifuged at 13,000 × *g* for 5 min at room temperature. The fluorescence intensity of the supernatant was measured using a Perkin Elmer 2030 Multilabel Reader with excitation at 420 nm and emission at 490 nm. The quantity of the reaction product, 1, 4-DMN, was calculated using a calibration curve at the linear range. NNMT activity was expressed in pmol/min/mg protein.

### Quantitation of the NAD^+^ metabolome

The samples were prepared, and targeted quantitative metabolomics was measured by LC/MS/MS as reported previously^15^ or with minor modifications. Briefly, methanol was added to plasma or urine and kidney homogenate for deproteinization and extraction. Each mixture was vortexed and centrifuged. The supernatant was diluted with water. Analyte separation and quantification were performed using a Shimadzu Nexera UHPLC system (Shimadzu, Kyoto, Japan) coupled with an API5000 triple quadrupole mass spectrometer (SCIEX, Framingham, MA, USA) with electrospray ionization operated in positive ion mode. The separation was performed on a Triart C18 column (3.0 × 150 mm, 5 μm, YMC, Kyoto, Japan). Quantification was performed by multiple reaction monitoring. NAD + , NADH, NADP, and NADPH in kidney tissue were measured using the NAD + /NADH Assay Kit (E2ND-100, EnzyChrom) and the NADP + /NADPH Assay Kit (ECNP-100, EnzyChrom), respectively.

### Bisulfite sequencing

Bisulfite sequencing was performed as previously reported^15^. Total kidney genomic DNA samples were extracted using GenElute Mammalian Genomic DNA Miniprep Kits (Sigma-Aldrich). Thereafter, samples were bisulfite-modified using an EpiTect Bisulfite Kit (Qiagen, Hilden, Germany). For bisulfite sequencing analysis, bisulfite-modified DNA samples were amplified using primers designed for the *CTGF CpG* island regions. The CTGF primer sequences were forward 5′-GTAGGTTTTATTAGTTT-3′; reverse 5′-CAAAAAAAACCCTTATATAAATC-3′. PCR products were cloned into pT7Blue T-vector, and positive clones were sequenced with an ABI 3130xl sequencer. The target sequences are shown in Supplementary Fig. 6A. Kikuchi et al. investigated the regulation of *CTGF* expression in human cell lines, and the methylation status around the *CTGF CpG* island correlated inversely with the expression, and the target region for methylation showed promoter activity^43^. Since there is a high degree of homology between mouse and human gene sequences in this region (Supplementary Fig. 6B), and the CpG island is also located in the same region, we conducted a methylation search using the above sequences, as previously reported^15^.

### Nuclei isolation and western blot analysis

The nuclei of kidney tissues were isolated using a nuclear protein isolation kit (Enzo Life Sciences). Samples with equal amounts of nuclear protein (10 μg) were used for western blot analysis. Primary antibodies against nuclear factor-kappa B (NF-κB) (1:2,000; ab16502) and Anti-NF-kB p65 (acetyl K310) (1:400; ab19870) were used. The blots were then incubated with a secondary antibody (1:5,000; Abcam) conjugated to horseradish peroxidase. Immunoreactive bands were detected using an ECL detection kit (Amersham Biosciences, Uppsala, Sweden). The lung tissue was used as a positive control for acetyl NF-κB, and HeLa cells were used as a positive control for total NF-κB, as indicated in the protocol for each antibody as shown in Supplementary Fig. 7.

### Patients with CKD

The ethics committee of Keio University approved the study protocol (Approval No. 20150340), and the study was conducted following the Declaration of Helsinki. Written informed consent was obtained from all patients. Patients diagnosed with CKD using the KDIGO 2012 Clinical Practice Guideline for the Evaluation and Management of Chronic Kidney Disease at the Keio University Hospital were included in the study^63^. The minimum number of samples required for analysis was calculated as 20 for each stage but was set at 30 (or at least 25) to allow for incomplete data, such as poor specimens. After excluding the samples with insufficient blood and urine data and infections, 139 patients were eventually included in the study. eGFR was calculated as average of eGFR [creatinine (Cr) = 194 × Serum creatinine (Scr)^-1.094^ × Age^-0.287^ × 0.739 (if female)] and eGFR [Cystatin C = {104 × (ScysC)^-1.019^ × 0.996^Age^ × 0.929 (if female)} -8], because it is closest to the measured GFR^64^. Medical history, medication use, body weight, body height, and blood pressure (BP) data were collected from medical records. Body mass index (BMI) was calculated as weight in kilograms divided by height in meters squared. Venous blood samples were collected to measure HbA1c, serum creatinine, serum cystatin C, and uric acid. Serum samples were stored in aliquots at − 80 ℃ for measurements of NAD + metabolites. Spot urine samples were collected to measure protein, albumin, and creatinine. Hypertension was defined as systolic BP ≥ 140 mm Hg, diastolic BP ≥ 90 mm Hg, or antihypertensive medication use^65,66^. Diabetes mellitus was defined by the use of current diabetes mellitus medication or HbA1c (National Glycohemoglobin Standardization Program) ≥ 6.5%, according to the International Expert Committee^67^. Hyperuricemia was defined as serum uric acid > 7.0 mg/dL, regardless of sex or age^68^.

### Renal biopsy

The review committee of Keio University approved the study protocol (Approval No. 20170005). Renal biopsies were performed as part of routine clinical diagnostic procedures at Keio University. We recruited patients who were judged to need a kidney biopsy in our clinical practice. Written informed consent was obtained for the biopsy (20 patients), but one patient was excluded because of an inadequate sample. Clinical characteristics of the study participants are shown in Supplementary Table 3. The biopsy samples were divided into two groups according to NNMT expression status, as evaluated by immunohistochemistry (IHC) (high group, *n* = 10; low group, *n* = 9). Positive areas for IHC staining and area of the fibrotic lesions (marked blue by Masson Goldner staining) were calculated with ImageJ software (http://rsbweb.nih.gov/ij/)^69^.

### Immunohistochemistry

In the mouse models, formalin-fixed and paraffin-embedded renal tissue sections were used for histological analysis. Masson-trichrome staining was performed to assess interstitial fibrosis (areas stained in blue), which was quantified using analysis software (ImageJ software) as described previously^70^. In addition, the invasion of macrophages was detected using primary antibodies against F4/80. Quantitative analysis of the number of cells positive for F4/80 was performed using the StrataQuest software, as described previously^71^. To evaluate oxidative stress, 4-HNE staining (NIKKEN SEIL) of renal tissue was performed, and the stained area in random 20 fields of view was evaluated using StrataQuest.

Human renal tissues were collected by needle biopsy and fixed in 10% neutral buffered formalin for 24 h and embedded in paraffin. Tissue sections were deparaffinized in xylene and rehydrated in ethanol. Sections were incubated with rabbit anti-human NNMT (1:200) (Proteintech). An EnVision™ + system (Dako) and 3, 3-diaminobenzidine (Roche) were utilized for visualization. Masson Goldner staining was conducted using a commercial kit (Merck)^72^.

### Statistical analysis

We used JMP 14.2 (SAS Institute, Cary, NC, USA) for statistical analyses. In the animal model, data are expressed as the mean ± SEM and were analyzed by the Student’s *t*-test for two groups and the Tukey–Kramer test for four groups. In human clinical research, data are presented as medians (25th and 75^th^ percentiles) for continuous variables or percentages for categorical variables. Continuous variable trends over five CKD stages were assessed using trend tests in a linear regression model. Linear trends of proportions were analyzed using the Cochrane-Armitage test for proportion trends^73^. Associations between eGFR and NAD + metabolites were examined using simple linear regression analyses. We then performed multiple linear regression analyses to evaluate the explanatory variable for each NAD + metabolite. If an association was strong, for example, between current body weight and BMI, we excluded one factor from the candidate confounders. In addition, we considered age, HbA1c, eGFR, and BMI during the multiple linear regression analysis, based on the previous reports^10,14,74^. The associations between NAD + metabolites and potential confounders/mediators were examined using multiple linear regression analysis. Multicollinearity was assessed using the variance inflation factor (VIF), and candidates had a VIF < 5.

## Supplementary Information

Supplementary Information.

## Data Availability

The data that support the findings of this study are available from the corresponding authors upon request.
