# Supplementary material for: The significance of NAD + metabolites and nicotinamide N-methyltransferase in chronic kidney disease
Source: Sci Rep. 2022 Apr 16;12:6398. doi: 10.1038/s41598-022-10476-6 (PMC9013399; doi:10.1038/s41598-022-10476-6)
Supplement: Supplementary file 1 — Supplementary Information. [file 41598_2022_10476_MOESM1_ESM.pdf]

The significance of NAD+ metabolites and Nicotinamide N-methyltransferase in chronic kidney disease

Rina Takahashi[1+], Takeshi Kanda[1+], Motoaki Komatsu[1], Tomoaki Itoh[1], Hitoshi Minakuchi[1], Hidenori Urai[1], Tomohiro Kuroita[2], Shuhei Shigaki[2], Tasuku Tsukamoto[3], Naoko Higuchi[3], Minoru Ikeda[3], Risa Yamanaka[3], Norito Yoshimura[3], Takashi Ono[3], Hideo Yukioka[3], Kazuhiro Hasegawa[1], Hirobumi Tokuyama[1], Shu Wakino[1]*, and Hiroshi Itoh[1]

Supplementary Table 1. Clinical Characteristics of the Study Subjects.

Characteristics	CKD						p-value for trend
	G1 (n = 25)	G2 (n = 30)	G3 (n = 32)	G4 (n = 26)	G5 (n = 26)	all stages (n = 139)	
age, y	38 (28–50)	66 (56–74)	72.5 (68–81)	78 (69–84)	69 (64–78)	69 (57–78)	< 0.01
male, n (%)	11(44)	14(47)	25(78)	20(77)	15(58)	85(61)	0.053
Body mass index (kg/m^2)	20.6 (19–25)	23.5 (22–27)	24.5 (22–27)	24.3 (23–27)	23.3 (20–27)	23.6 (21–26)	0.19
eGFR (mL/min/1.73 m^2)	104 (99–115)	76.5 (68–83)	47.0 (40–54)	22.5 (19–27)	10.5 (9–13)	47.0 (20–83)	< 0.01
hypertension, n (%)	11(44)	26(87)	27(84)	21(81)	25(96)	110(79)	< 0.01
diabetes mellitus, n (%)	4(16)	5(17)	9(28)	9(35)	11(42)	38(27)	< 0.01
hyperuricemia, n (%)	2(8)	7(23)	19(59)	21(81)	24(92)	73(53)	< 0.01
HbA1c (%)	5.5 (5.4–5.9)	5.8 (5.5–6.2)	6.0 (5.6–6.2)	6.0 (5.7–6.3)	5.8 (5.4–6.1)	5.8 (5.5–6.2)	0.54
UA (mg/dL)	4.4 (4.2–5.3)	5.9 (5.1–6.9)	6.3 (5.7–6.9)	6.8 (6.2–7.2)	6.6 (5.3–7.6)	6.1 (5.0–6.9)	< 0.01
Proteinuria (g/gCr)	0.11 (0.04–0.77)	0.21 (0.07–0.55)	0.16 (0.08–0.67)	0.56 (0.29–2.47)	2.04 (0.78–5.22)	0.43 (0.10–1.60)	< 0.01
Albuminuria (mg/gCr)	20.9 (5.7–183)	85.8 (15.4–290)	37.6 (12.2–388)	323 (92.0–1,559)	1,086 (315–2,916)	172 (16.8–83)	< 0.01

Data are shown as medians with interquartile range or number with the percentage in parentheses. eGFR, estimated glomerular filtration rate. The definitions of hypertension, diabetes mellitus, and hyperuricemia are shown in the Methods. All p-values are for trends.

Supplementary Table 2. Multiple regression analysis showing independent contributions to NAD+ metabolites.

Serum NAM: Whole-model adjusted $R^2 = 0.0435$, $p < 0.05$

Parameters	estimate	SE	β	P
Intercept	68.3	52.3		0.19
Age	-0.058	0.34	-0.018	0.87
HbA1c	4.4	8.4	0.048	0.6
BMI	0.46	1.2	0.036	0.69
eGFR	0.41	0.17	0.26	< 0.05

Serum 2-PY: Whole-model adjusted $R^2 = 0.332$; $p < 0.01$

Parameters	Estimate	SE	β	P
Intercept	14,000	3,800		< 0.01
Age	-65	25	-0.24	< 0.05
HbA1c	210	610	0.027	0.73
BMI	-79	84	-0.072	0.35
eGFR	-98	13	-0.72	< 0.01

Serum 4-PY: Whole-model adjusted $R^2 = 0.332$, $p < 0.01$

Parameters	Estimate	SE	β	P
Intercept	2,300	670		< 0.01
Age	-9.3	4.4	-0.2	< 0.05
HbA1c	41	110	0.03	0.7
BMI	-12	15	-0.063	0.42
eGFR	-17	2.2	-0.71	< 0.01

Urinary NAM: Whole-model adjusted $R^2 = 0.0793$, $p < 0.01$

Parameters	Estimate	SE	β	P
Intercept	0.51	0.41		0.22
Age	-0.003	0.0027	-0.12	0.27
HbA1c	0.012	0.065	0.017	0.85
BMI	-0.0067	0.009	-0.066	0.46
eGFR	0.0027	0.0013	0.22	< 0.05

Variables expected to influence NAD+ metabolites levels (eGFR, HbA1c, age, and BMI) were included in the models.

Supplementary Table 3. Clinical characteristics of patients used in the renal histologic study

Pt No	age	sex	Cr (mg/dL)	eGFR (mL/min /1.73 m^2)	BMI	u-Alb (mg/gCr)	u-Prot (g/gCr)	Hema turia	HTN	DM	HbA1c (%)	primary disease
1	65	F	0.84	52	22.3	547	0.8	2+	+	-	5.5	minimal change
2	55	M	3.11	18	22.2	1,784	2.9	±	+	-	6.5	IgA nephropathy
3	29	M	1.58	45	18.3	878	1.3	-	-	-	5.4	IgA nephropathy
4	61	M	1.93	29	23.2	713	1.1	3+	+	-	6.4	IgA nephropathy
5	38	M	1.67	39	19.9	853	1.2	3+	+	-	5.6	IgA nephropathy
6	43	M	0.69	99	23	538	0.9	3+	+	-	4	IgA vasculitis
7	31	F	0.61	91	17.9	35	0.1	-	+	-	5.1	membranous nephropathy
8	66	M	2.15	25	20.6	5,748	8.4	1+	+	-	5.2	membranous nephropathy
9	57	M	1.16	52	28.7	1,331	1.9	3+	-	+	5.8	PGNMID
10	53	M	0.77	83	23.3	4,571	6.7	1+	+	+	7.5	membranous nephropathy
11	60	M	1.36	43	21.4	519	0.8	-	+	-	4.7	membranous nephropathy
12	45	M	2.29	26	39.3	1,823	3.1	3+	+	+	6.7	adaptive FSGS
13	61	M	1.05	57	24.1	5	0.1	-	-	+	6.9	minimal change
14	70	M	1.33	42	28.4	1,447	2.5	3+	+	-	5	adaptive FSGS
15	50	F	0.59	83	31.9	1,007	1.5	-	+	-	5.6	membranous nephropathy
16	56	F	1.13	40	20.7	151	0.8	-	-	-	5.3	Fanconi synd
17	69	M	1.1	52	27.4	314	0.5	-	+	+	6.4	adaptive FSGS
18	50	M	0.95	67	24.6	389	0.7	1+	+	+	6.8	minimal change
19	59	M	2.13	26	25.2	2,989	4.6	+-	+	+	6.7	Diabetic nephropathy

F; female, M; male. Cr; creatinine, eGFR; estimated glomerular rates, u-Alb; urinary albumin excretion, u-Prot; urinary protein excretion, HTN; hypertension, DM; diabetes, BMI; body mass index, PGNMID; proliferative glomerulonephritis with monoclonal IgG deposits, FSGS; Focal segmental glomerulosclerosis.

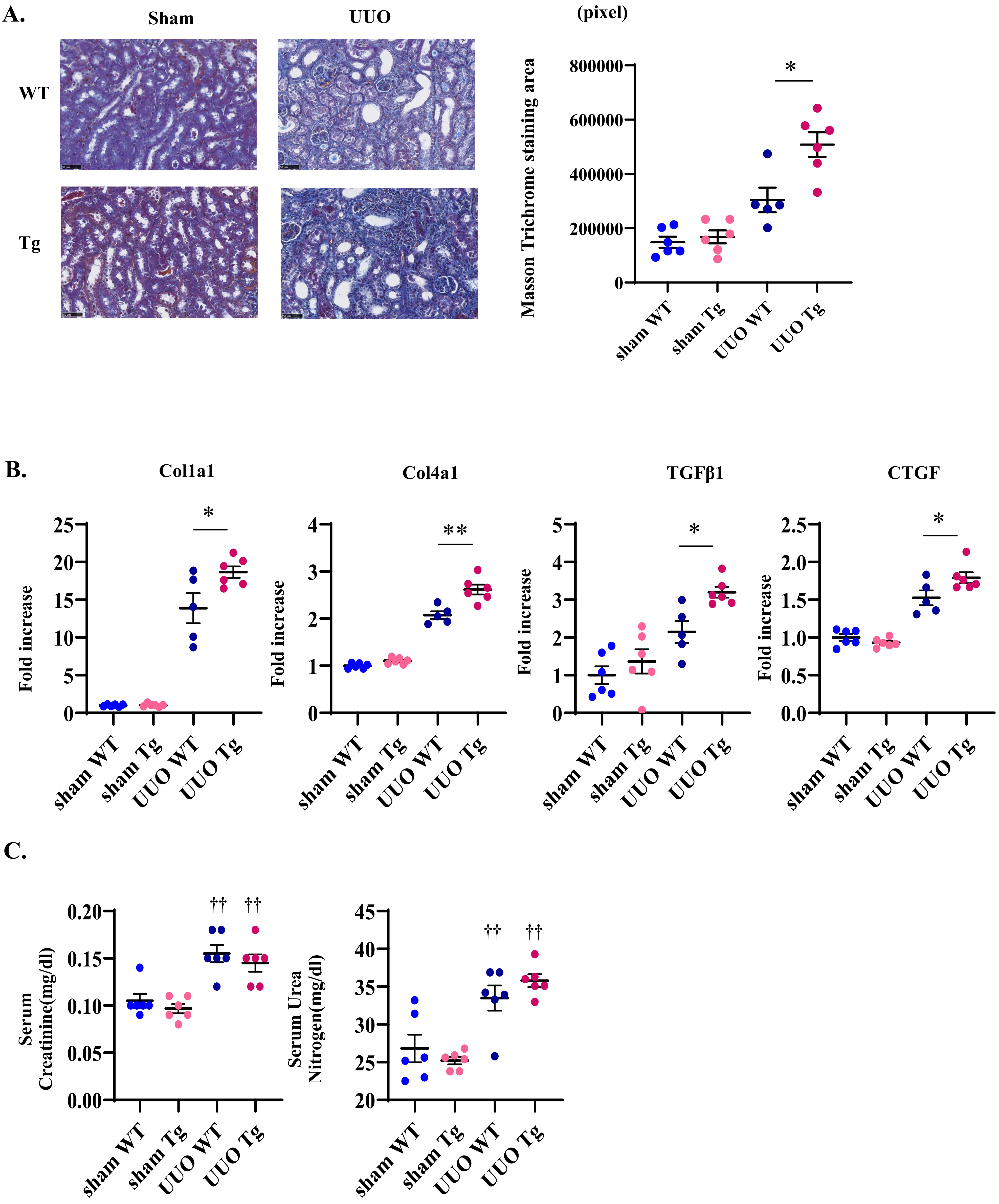

Supplementary Fig. 1, Takahashi et al.

A.

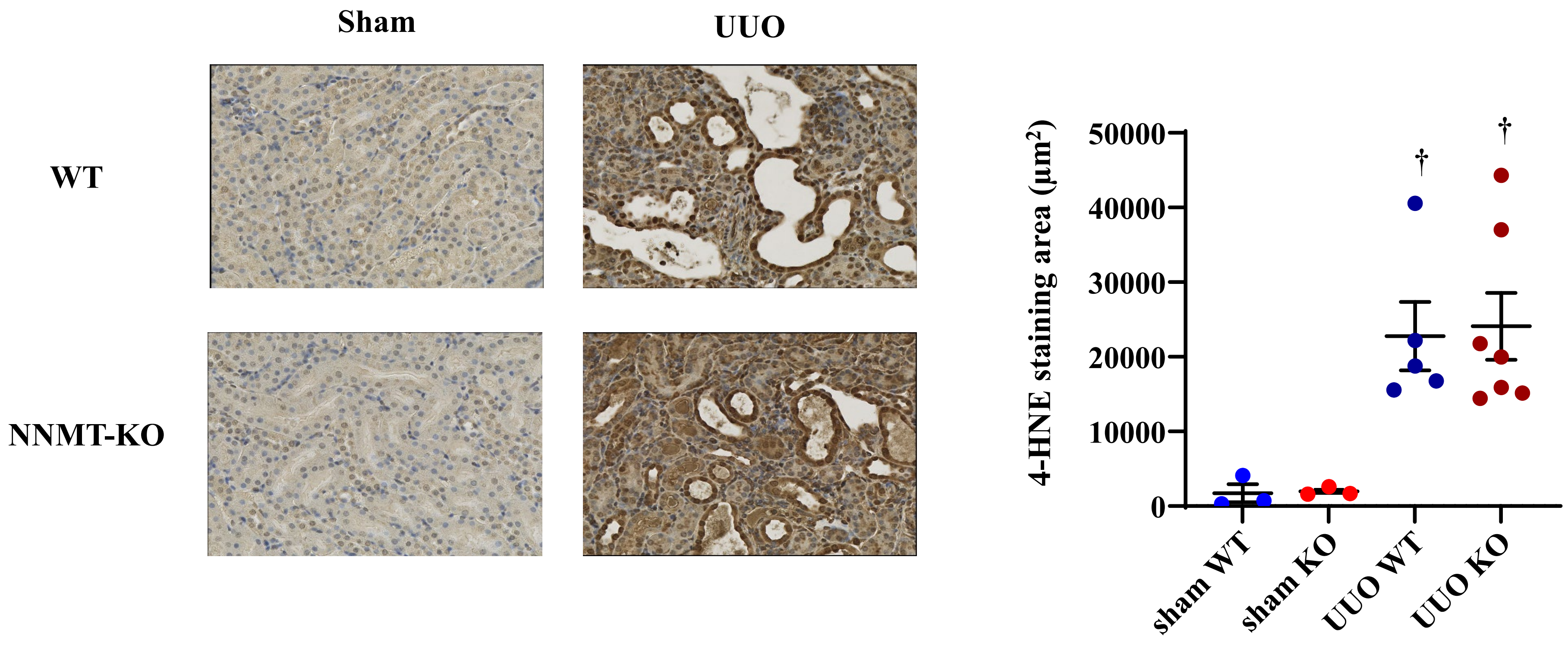

B.

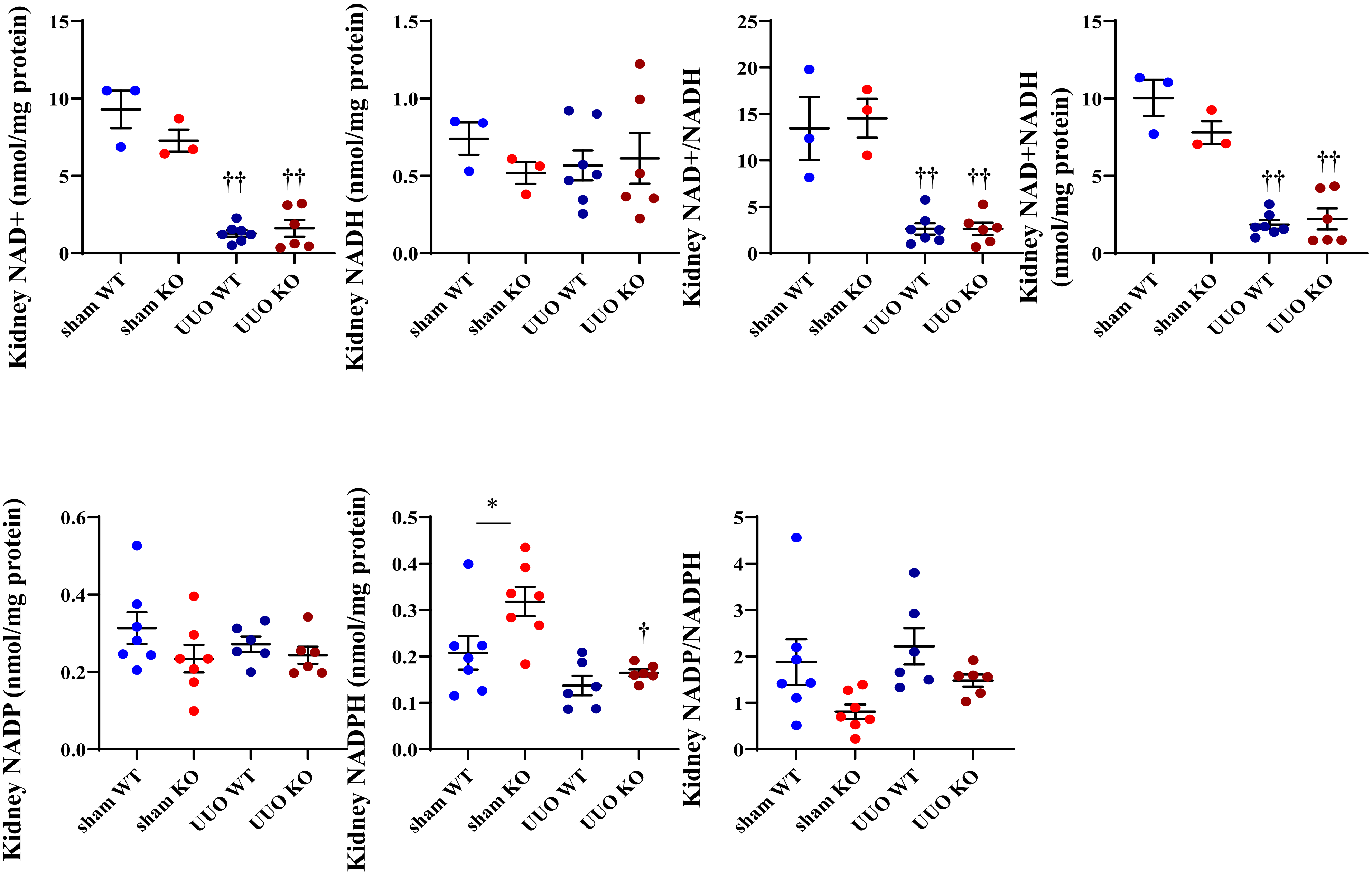

Supplementary Fig. 2, Takahashi et al.

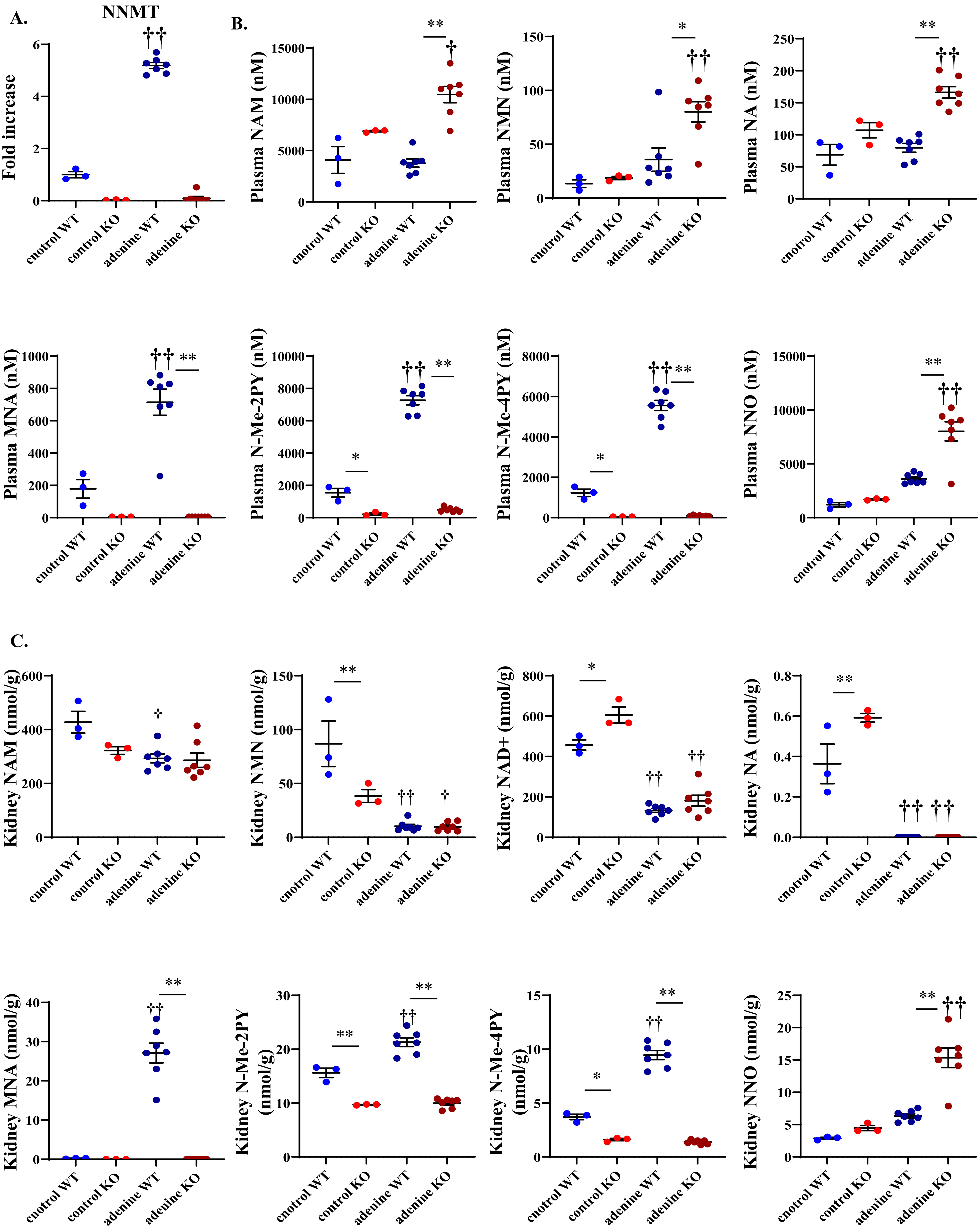

Supplementary Fig. 3, Takahashi et al.

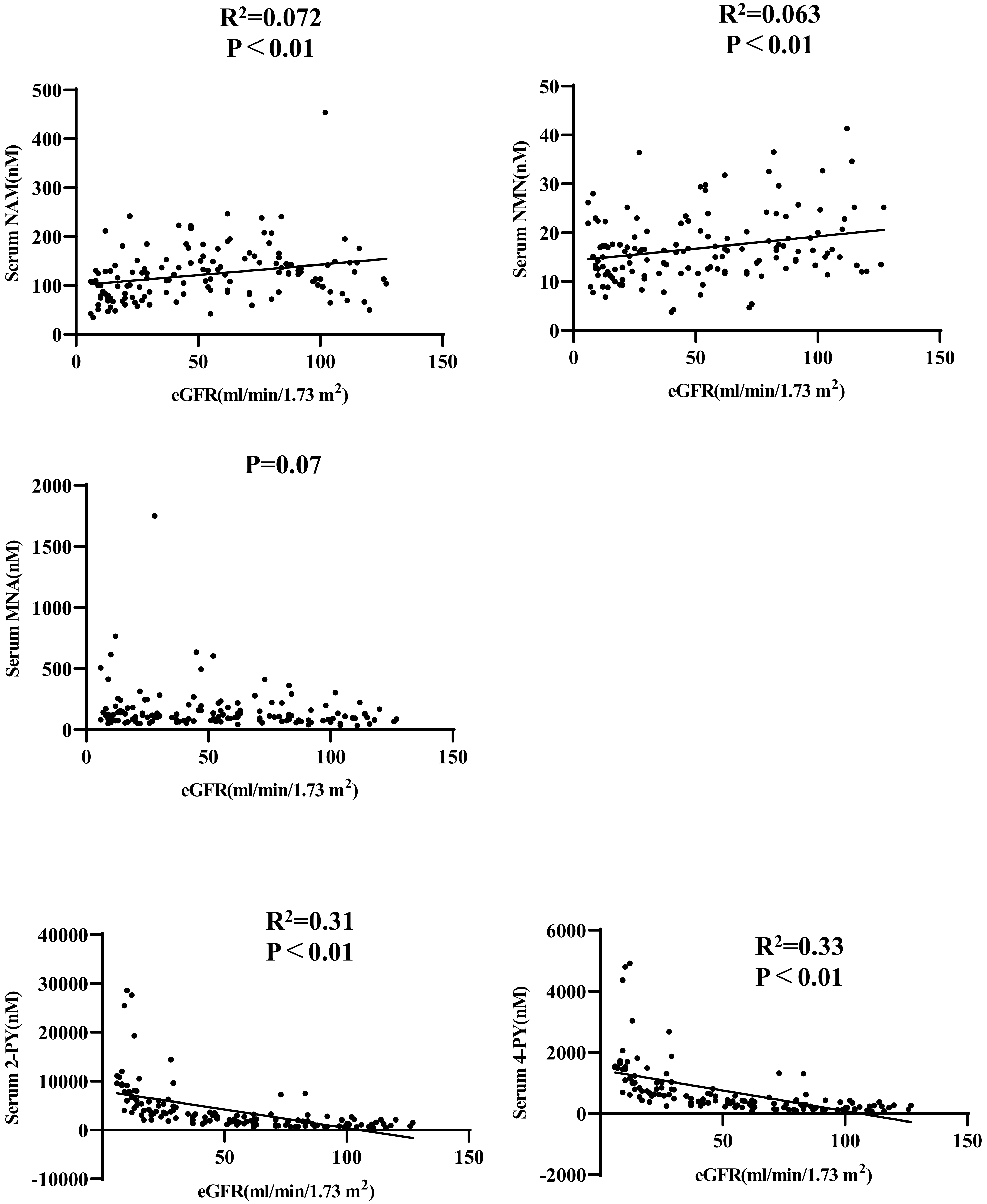

Supplementary Fig. 4, Takahashi et al.

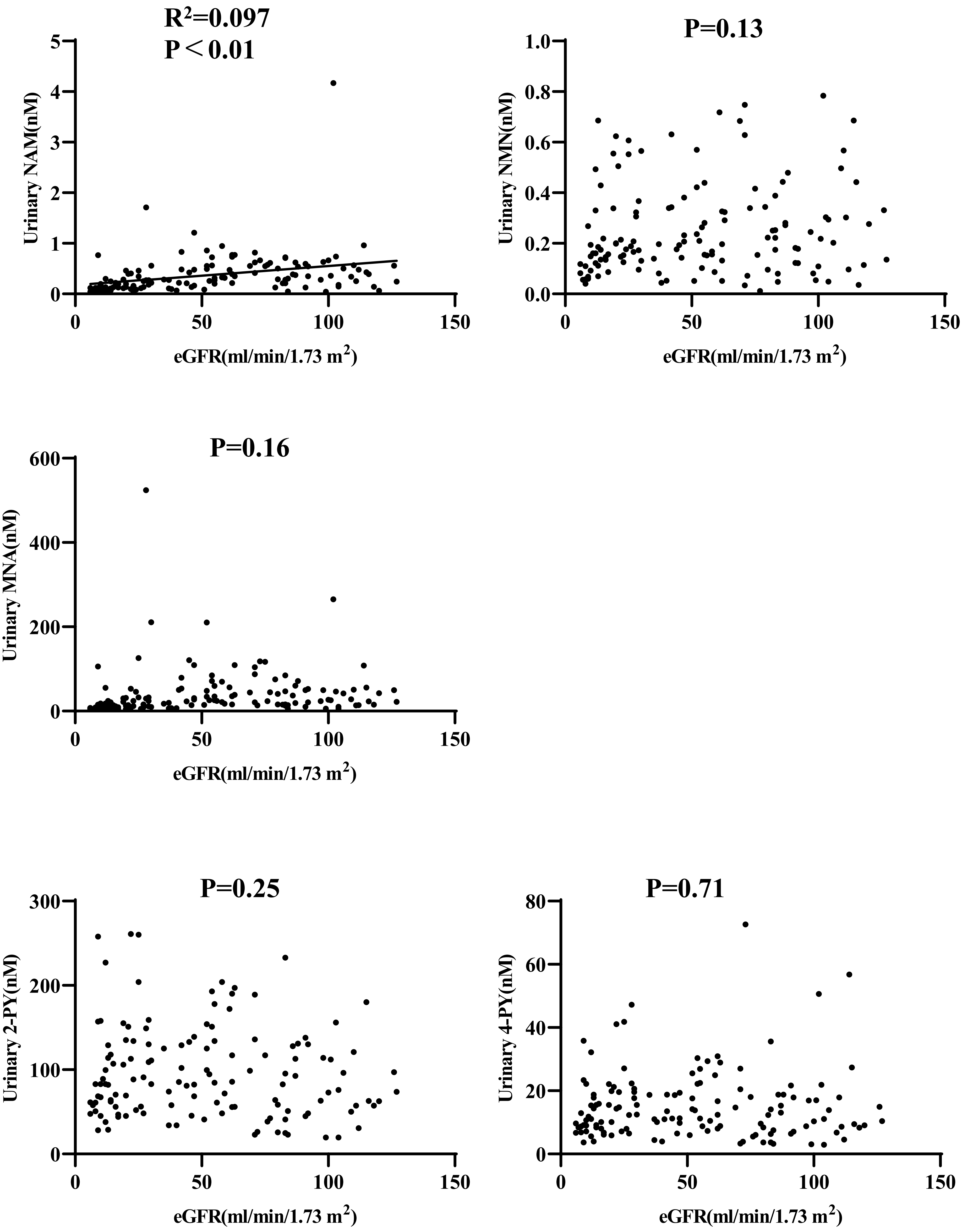

Supplementary Fig. 5, Takahashi et al.

A.

Bisulfite sequencing analysis area (Ctgf gene)

Black shaded area: Analysis area (chr10:24,595,758-24,595,990)
Yellow area: CG sequence
Black shaded area and yellow area: target CG sequence (22 locations)
enclosure line: Primer design position

Before Bisulfite treatment

```
  1 GTGGAGTGTC AAGGGGTCAG GATCAATCCG GTGTGAGTTG ATGAGGCAGG AAGGTGGGGA
                                 Exon1 (NM_010217) 5' UTR region
 61 GGAATGTGAG GAATGTCCCT GTTTGTGTAG GACTCCATTC AGTTCTTTGG CGAGCCGGCT
121 CCCGGGAGCG TATAAAAGCC AGCGCCGCCC GCCTAGTCTC ACACAGCTCT TCTCTCCAAG
181 AAGACTCAGC CAGATCCACT CCAGCTCCGA CCCCAGGAGA CCGACCTCCT CCAGACGGCA
241 GCAGCCCCAG CCCAGCCGAC AACCCCAGAC GCCACCGCCT GGAGCGTCCA GACACCAACC
301 TCCGCCCCTG TCCGAATCCA GGCTCCGGCC GCGCCTCTCG TCGCCTCTGC ACCCTGCTGT
                         Initiation codon ORF                    1
361 GCATCCTCCT ACCGCGTCCC GATCATGCTC GCCTCCGTCG CAGGTCCCAT CAGCCTCGCC
                 2          3           4         5         6
421 TTGGTGCTCC TCGCTCTCTG CACCCGGGTA AGCCCCGGGA CTGACGGAAG GGACGGAGGG
                     7                                 8
481 AGGGCAGAGT GAGCTGCGAT CACAGACTGA CCTCCCTCCC CTTCCTCTCC GCAGCCTGCT
                     9 10          11     12       13 14         15
541 ATGGGCCAGG ACTGCAGCGC GCAATGTCAG TGCGCAGCCG AAGCAGCGCC GCACTGCCCC
      16 17                    18     19       20 21     22
601 GCCGGCGTGA GCCTGGTGCT GGACGGCTGC GGCTGCTGCC GCGTCTGCGC CAAGCAGCTG
661 GGAGAACTGT GTACGGAGCG TGACCCCTGC GACCCACACA AGGGCCTCTT CTGCGATTTC
721 GGCTCCCCCG CCAACCGCAA GATCGGAGTG TGCACTGGTA AGACCCTCAG CCCCATTCCA
```

After Bisulfite treatment

※Lowercase t is the sequence in which unmethylated C was converted

```
  1 GTGGAGTGTt AAGGGGTtAG GATtAATtCG GTGTGAGTTG ATGAGGtAGG AAGGTGGGGA
                                 Exon1 (NM_010217) 5' UTR region
 61 GGAATGTGAG GAATGTtttT GTTTGTGTAG GAtTttATTt AGTTtTTTGG CGAGtCGGtT
121 ttCGGGAGCG TATAAAAGtt AGCGtCGttC GttTAGTtTt AtAtAGtTtT TtTtTttAAG
181 AAGAtTtAGt tAGATttAtT ttAGtTtCGA ttttAGGAGA tCGAttTttT ttAGACGGtA
241 GtAGttttAG tttAGtCGAt AAttttAGAC GttAtCGttT GGAGCGTttA GAtAttAAtt
301 TtCGttttTG TtCGAATttA GGtTtCGGtC GCGttTtTCG TCGttTtTGt AtttTGtTGT
                           Initiation codon ORF Forward Primer   1
361 GtATttTttT AtCGCGTttC GATtATGtTC GttTtCGTCG tAGGTtttAT tAGttTCGtt
                 2          3           4         5         6
421 TTGGTGtTtt TCGtTtTtTG tAttCGGGTA AGtttCGGGA tTGACGGAAG GGACGGAGGG
                     7                                 8
481 AGGGtAGAGT GAGtTGCGAT tAtAGAtTGA ttTtttTttt tTTttTtTtC GtAGttTGtT
                     9 10          11     12       13 14         15
541 ATGGGttAGG AtTGtAGCGC GtAATGTtAG TGCGtAGtCG AAGtAGCGtC GtAtTGtttC
      16 17                    18     19       20 21     22
601 GtCGGCGTGA GttTGGTGtT GGACGGtTGC GGtTGtTGtC GCGTtTGCGt tAAGtAGtTG
                                   Reverse Primer
661 GGAGAAtTGT GTACGGAGCG TGAttttTGC GAtttAtAtA AGGGttTtTT tTGCGATTTt
721 GGtTttttCG ttAAtCGtAA GATCGGAGTG TGtAtTGGTA AGAtttTtAG ttttATTttA
```

Supplementary Fig. 6, Takahashi et al.

B.

Sequence containing 200 bp before and after the Coding region of Exon1
Pink: the Coding region of Exon1

・Region around the start codon of the mouse Ctgf gene (466 bp)

```
  1 CTCAGCCAGA TCCACTCCAG CTCCGACCCC AGGAGACCGA CCTCCTCCAG ACGGCAGCAG
 61 CCCCAGCCCA GCCGACAACC CCAGACGCCA CCGCCTGGAG CGTCCAGACA CCAACCTCCG
121 CCCCTGTCCG AATCCAGGCT CCGGCCGCGC CTCTCGTCGC CTCTGCACCC TGCTGTGCAT
181 CCTCCTACCG CGTCCCGATC ATGCTCGCCT CCGTCGCAGG TCCCATCAGC CTCGCCTTGG
241 TGCTCCTCGC TCTCTGCACC CGGGTAAGCC CCGGGACTGA CGGAAGGGAC GGAGGGAGGG
301 CAGAGTGAGC TGCGATCACA GACTGACCTC CCTCCCCTTC CTCTCCGCAG CCTGCTATGG
361 GCCAGGACTG CAGCGCGCAA TGTCAGTGCG CAGCCGAAGC AGCGCCGCAC TGCCCCGCCG
421 GCGTGAGCCT GGTGCTGGAC GGCTGCGGCT GCTGCCGCGT CTGCGC
```

・Region around the start codon of the human CTGF gene (466 bp)

```
  1 ACACAACAAC TCTTCCCCGC TGAGAGGAGA CAGCCAGTGC GACTCCACCC TCCAGCTCGA
 61 CGGCAGCCGC CCCGGCCGAC AGCCCCGAGA CGACAGCCCG GCGCGTCCCG GTCCCCACCT
121 CCGACCACCG CCAGCGCTCC AGGCCCCGCC GCTCCCCGCT CGCCGCCACC GCGCCCTCCG
181 CTCCGCCCGC AGTGCCAACC ATGACCGCCG CCAGTATGGG CCCCGTCCGC GTCGCCTTCG
241 TGGTCCTCCT CGCCCTCTGC AGCCGGGTAA GCGCCGGGAG CCCCCGCTGC GGCCGGCGGC
301 TGCCAGGGAG GGACTCGGGG CCGGCCGGGG AGGGCGTGCG CGCCGACCGA GCGCCGCTGA
361 CCGCCCTGTC CTCCCTGCAG CCGGCCGTCG GCCAGAACTG CAGCGGGCCG TGCCGGTGCC
421 CGGACGAGCC GGCGCCGCGC TGCCCGGCGG GCGTGAGCCT CGTGCT
```

Supplementary Fig. 6, Takahashi et al.

acetyl NF-κB

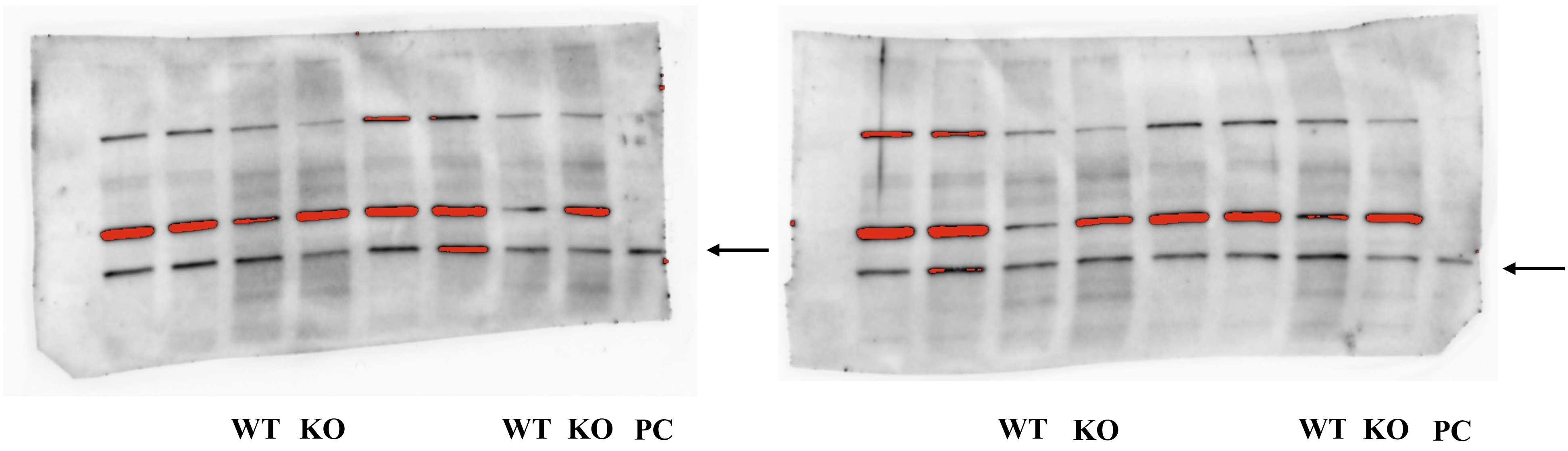

Total NF-κB

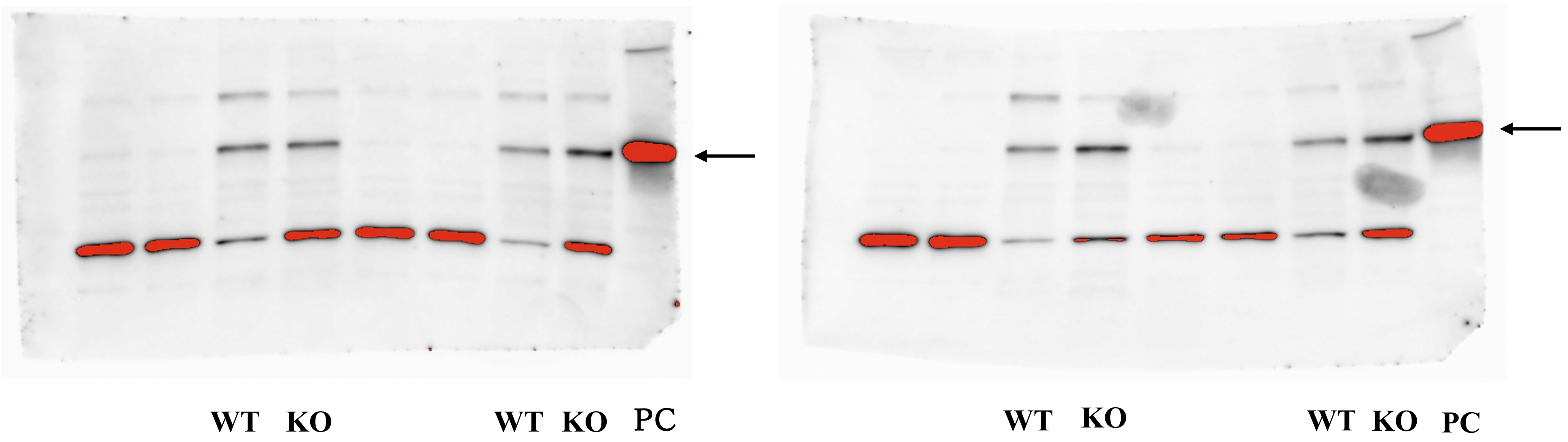

Supplementary Fig. 7, Takahashi et al.

Supplementary figure legends

Supplementary Fig. 1. Overexpression of NNMT deteriorates renal fibrotic changes in the UUO model.

(A) Renal fibrosis was assessed by Masson-trichrome staining in the kidneys of NNMT-Tg mice and WT littermates after UUO induction. The data represent means ± SEM (n = 5–6 per group). (B) Fibrosis-related genes were measured in the kidneys of NNMT-Tg mice and WT littermates after UUO induction. The data represent means ± SEM (n = 5–6 per group). (C) Renal function (serum creatinine and urea nitrogen) was measured in NNMT-KO mice and WT littermates after UUO induction. The values shown are means ± SEM (n = 6 per group).

UUO, unilateral ureter obstruction; NNMT, nicotinamide N-methyltransferase; Col, collagen; CTGF, connective tissue growth factor; TGF, transforming growth factor. *$p < 0.05$, **$p < 0.01$ versus WT mice.

Supplementary Fig. 2. No significant change in oxidative stress was observed upon NNMT suppression in UUO.

(A) Oxidative stress was assessed by 4-HNE staining in the kidneys of NNMT-KO mice and WT littermates after UUO induction. The data is represented as mean ± SEM (n = 3–7 per group). (B) NAD(P)(H) was measured in the kidneys of NNMT-KO mice and WT littermates after UUO induction. The data is represented as mean ± SEM (n = 3–7 per group).

4-HNE, 4-hydroxy-2-nonenal; NAD, nicotinamide adenine dinucleotide; NADP, nicotinamide adenine dinucleotide phosphate.

Supplementary Fig. 3. Both adenine and UUO models show similar changes in NNMT expression and NAD metabolites in serum and renal tissues.

(A) NNMT mRNA expression was measured in NNMT-KO mice and WT littermates in the adenine model. The values are shown as mean ± SEM (n = 3–7 per group). Plasma NAD+ metabolites (B) and renal NAD+ metabolites (C) were measured in NNMT-KO mice and WT littermates in the adenine model. The values are shown as mean ± SEM (n = 3–7 per group).

NNMT, nicotinamide N-methyltransferase; NAM, nicotinamide; NMN, nicotinamide mononucleotide; NAD+, nicotinamide adenine dinucleotide; MNA, 1-methylnicotinamide; N-Me-2PY, N-methyl-2-pyridone-5-carboxamide; N-Me-4PY, N-methyl-4-pyridone-3-carboxamide; NA, nicotinic acid; NNO, nicotinamide-N-oxide.

Supplementary Fig. 4. Serum NAM and NMN concentration are declined, while 2-PY and 4-PY are elevated in parallel with reduced eGFR.

Correlation between serum NAD+ metabolites and eGFR.

eGFR, estimated glomerular filtration rate; NAM, nicotinamide; NMN, nicotinamide mononucleotide; MNA, 1-methylnicotinamide; 2-PY, N-methyl-2-pyridone-5-carboxamide; 4-PY, N-methyl-4-pyridone-3-carboxamide.

Supplementary Fig. 5. Urinary NAM concentration is declined in parallel with reduced eGFR.

Correlation between urinary NAD+ metabolites and eGFR.

eGFR, estimated glomerular filtration rate; NAM, nicotinamide; NMN, nicotinamide mononucleotide; MNA, 1-methylnicotinamide; 2-PY, N-methyl-2-pyridone-5-carboxamide; 4-PY, N-methyl-4-pyridone-3-carboxamide.

Supplementary Fig. 6. Target Bisulfite sequences used and DNA homology between mice and humans at the site.

(A) Target sequences identified from Bisulfite sequencing and (B) DNA homology between mice and humans at the site.

Supplementary Fig. 7. Full-length blots/gels of Figure 5F.

Full-length blots/gel of western blotting of acetyl and total NF- κ B are presented.
